# DynaFace: Discrimination between Obligatory and Non-obligatory Protein-Protein Interactions Based on the Complex’s Dynamics

**DOI:** 10.1371/journal.pcbi.1004461

**Published:** 2015-10-27

**Authors:** Seren Soner, Pemra Ozbek, Jose Ignacio Garzon, Nir Ben-Tal, Turkan Haliloglu

**Affiliations:** 1 Department of Computer Engineering and Polymer Research Center, Bogazici University, Istanbul, Turkey; 2 Department of Bioengineering, Marmara University, Istanbul, Turkey; 3 Departments of Biochemistry and Molecular Biophysics and Systems Biology and Howard Hughes Medical Institute, Columbia University, New York, New York, United States of America; 4 Department of Biochemistry and Molecular Biology, George S. Wise Faculty of Life Sciences, Tel Aviv University, Tel Aviv, Israel; 5 Department of Chemical Engineering and Polymer Research Center, Bogazici University, Istanbul, Turkey; Bar Ilan University, ISRAEL

## Abstract

Protein-protein interfaces have been evolutionarily-designed to enable transduction between the interacting proteins. Thus, we hypothesize that analysis of the dynamics of the complex can reveal details about the nature of the interaction, and in particular whether it is obligatory, i.e., persists throughout the entire lifetime of the proteins, or not. Indeed, normal mode analysis, using the Gaussian network model, shows that for the most part obligatory and non-obligatory complexes differ in their decomposition into dynamic domains, i.e., the mobile elements of the protein complex. The dynamic domains of obligatory complexes often mix segments from the interacting chains, and the hinges between them do not overlap with the interface between the chains. In contrast, in non-obligatory complexes the interface often hinges between dynamic domains, held together through few anchor residues on one side of the interface that interact with their counterpart grooves in the other end. In automatic analysis, 117 of 139 obligatory (84.2%) and 203 of 246 non-obligatory (82.5%) complexes are correctly classified by our method: DynaFace. We further use DynaFace to predict obligatory and non-obligatory interactions among a set of 300 putative protein complexes. DynaFace is available at: http://safir.prc.boun.edu.tr/dynaface.

## Author Summary

Protein-protein interactions mediate, in essence, all inter- and intra-cellular processes. Thus, understanding their molecular mechanism is of utmost importance. Here we focus on one mechanistic aspect: differentiation between obligatory interactions, which persist throughout the entire lifetime of the protein complex, and non-obligatory, which do not. For proper function, a protein complex should facilitate transduction between the interacting proteins. Therefore the complex’s dynamics should reveal whether it is obligatory or non-obligatory. Indeed, normal mode analysis shows that the dynamic domains of obligatory complexes often mix segments from the interacting chains. In contrast, in non-obligatory complexes the inter-chain interface often hinges between dynamic domains, held together through few anchor residues. An automated methodology based on these observations correctly classifies over 80% of the interfaces in a test set. We use it also to predict obligatory and non-obligatory interactions among putative protein complexes. DynaFace, a web-server implementation of the methodology, is available at: http://safir.prc.boun.edu.tr/dynaface.

“This is a *PLOS Computational Biology* Methods paper”

## Introduction

Inter-protein interactions mediate a wide range of cellular and biochemical processes [1, 2]. The Protein Data Bank (PDB; [3]) includes a wealth of information on these important interactions, observed in X-ray crystal, NMR, and cryo-electron microscopy structures. Indeed, this large resource has been exploited to deduce interactions [4, 5], and infer interactions based on similarity in sequence and/or structure [6–8]. It is noteworthy, however, that the PDB includes various types of interactions, many of which are physiologically irrelevant and reflect crystal packing [9–11]. Here we focus on the rest, i.e., physiologically realistic interactions, and differentiate between these that are obligatory and non-obligatory.

Protein chains engaged in an obligatory complex are found only in association with their partner chains and bind throughout their functional lifetime, for example, because they are unstable on their own [12]. The most popular example here is the interaction between the beta and gamma subunits of G-proteins, which remain intact throughout their lifetime. In contrast, non-obligatory complexes, such as the interaction between the beta-gamma complex and the alpha subunit of the G-protein, form and dissociate in response to environmental changes. These proteins are stable both in their bound and unbound states, although their conformation may change upon binding. They are abundant, for example, in signal transduction, antibody-antigen interactions, and enzyme-inhibitor complexes. The obligatory-vs.-not classification is not always straightforward. For example, at least theoretically, one chain might be unstable alone and gain stability only upon association with another chain, which in turn is stable also alone. Furthermore, a chain with two domains might be engaged in obligatory interaction through the first domain and a non-obligatory interaction through the second, which again complicates the classification [13]. Additionally, the nature of the interaction may alter in response to external changes, such as pH, temperature, interaction with ligand, etc.

Non-obligatory interactions could be further classified as transient or permanent, depending on their lifetimes, providing a kinetic dimension of the association. In addition, changes in pH, ionic strength or concentrations of the interacting chains can shift the dynamic equilibrium towards or away from association. Thus, transient complexes can be further classified as “weak” or “strong”, which are generally found in their unbound and bound states, respectively [12].

Obligatory interfaces compared to non-obligatory interfaces are larger, flatter and more evolutionarily conserved [14, 15]. Residues involved in obligatory interactions evolve at a slower rate, while residues in non-obligatory interactions exhibit an increased rate of substitution for faster adaptation required. Obligatory interfaces have higher shape complementary consisting primarily of side-chain contacts and larger interface-to-surface ratio [14]. On the other hand, non-obligatory interfaces are usually smaller and more polar (except enzyme-inhibitor complexes) with lower geometric complementary and weaker association where backbone plays an important role [16]. The latter features should provide the interfaces with optimum topological means for the required functional motion and interaction.

Previous studies have focused mostly in the analysis and prediction of protein surfaces that mediate protein-protein interactions rather than in differentiation between the interaction types. Evolutionary conservation profiles of the amino acid positions have appeared as a valuable source of information for the success of sequence-based methods in binding site predictions [17–20]. Structure-based methods, on the other hand, could make use of some additional properties, such as solvent accessible surface area and shape complementarity methods for more successful predictions [21–23]. Combined with structural information, the patches of conserved amino acids on protein surfaces were shown to have functional importance [24–26]. Recently developed machine learning methods using several attributes in the latter provide algorithms with plausible performances [22, 27, 28].

The prediction of protein interaction sites and/or interaction type based on a single property is challenging [22]. The existing web-servers use various sequence and structure properties alone and combined. For example, web-servers such as Promate [29], PPI-Pred [30, 31], Con-PPISP [32], meta-PPISP [30], PRISM [33], SPPIDER [34], IBIS [35] and metaPIS [36] focus on binding site predictions. Promate [29] uses surface properties with various physicochemical properties to predict transient interactions. PPI-Pred [31], Cons-PPISP [32], PRISM [33, 37] and SPPIDER [34] predict interfaces that may include both obligatory and non-obligatory interactions. PPI-Pred uses a support vector machine method in combination with surface patch analysis, and Cons-PPISP is a structure-based neural network method mainly using sequence profiles and solvent accessibilities. PRISM predicts interfaces by structural matching. SPPIDER is mainly based on solvent accessibility. IBIS uses conservation of sequence and structure for binding site predictions and metaPIS is mainly based on protein sequences. PrePPI combines structural modeling with other genomic, evolutionary and functional clues for the prediction of binary interactions [6]. For a query protein pair, representative structures of the subunits are first searched in the PDB and in homology model databases and then a search for structural neighbors follows. If two neighbors of each subunit are found in a complex in the PDB, then this complex is used as template. As a scoring value, individual subunits are superposed on the template complex and a likelihood ratio is calculated in combination with the non-structural naïve Bayesian classifier.

The NOXclass web-server [38] is unique in providing automatic classification of the interaction/interface types of query protein complexes. NOXclass is a support vector machine classifier making use of properties such as interface area and interface/surface area ratio, amino acid composition, shape complementarity and residue conservation for the interaction types. Alternatively, SCOPPI (Structural Classification of Protein-Protein Interfaces) [39] is a database that classifies the interface type by using knowledge on protein domain-domain interactions with known structures. The domain interactions are determined by a distance-based criterion and the domain definitions are obtained from SCOP [40], where proteins are classified based on both structural and evolutionary relatedness. The BindML+ web-server [41] predicts the interface type in a single protein as transient or permanent without the knowledge of its interacting partner. Here the definitions of non-obligatory but permanent might have been confused with obligatory interactions. PiType [42] is a downloadable program classifying protein interactions into simultaneously possible and mutually exclusive as well as into obligate and non-obligate based on the sequence and functional properties of the binding partners and their network context based on amino acid sequence and functional similarity.

To facilitate biological functionality, protein-protein association should involve transduction, e.g., of signal. Thus, it is anticipated that protein complexes that differ in their functionalities would manifest different dynamic behavior. In other words, the dynamic infrastructure underlying non-obligatory and obligatory interactions should be in compliance with the required functional motion of the respective interface types. Indeed, a recent analysis of proteins with multiple conformations in PDB showed that, on average, transient associations involve smaller conformational changes than permanent associations [43]. Here again, non-obligatory but permanent interfaces might be confused with obligatory interfaces. We attempt to take a step forward and try to classify obligatory vs. non-obligatory interfaces based on the analysis of dynamic fluctuations starting from a single conformation and link this with the functionality at the level of interaction types on a dataset of obligatory and non-obligatory interfaces. The dynamic fluctuations are calculated using the Gaussian Network Model (GNM) [44, 45], and used to differentiate between obligatory and non-obligatory interactions. We further analyze a set of structural models predicted using PrePPI [6].

## Methods

### Datasets

The dataset used here is a compilation of two available datasets of protein-protein interface types [14, 31]. Both datasets were compiled from the PDB [46] and their interfaces were then manually curated with the existing literature. The PISCES server [47] was used to reduce redundancy by removing proteins with over 25% sequence identity, resolution of 3.0 Å or better, and R factor of 0.3 or better. While combining the two datasets, some proteins were removed due to the redundancy and high number of missing residues or changes with the updates. After preliminary application of DynaFace on the set we noted cases of disagreement between prediction and annotation. Literature survey showed that the annotation of some of these was erroneous, presumably because of studies published after the compilation of the original sets [62–86]. These were fixed. The final dataset, consisting of 139 obligatory and 246 non-obligatory complex structures, is provided in S1 Table. The corrections are marked. It is noteworthy that the set includes 84 multi-subunit complexes. For these, at least one of the interacting units includes more than one polypeptide chain.

Additionally, a dataset of predicted structural models [6] is used to make testable hypotheses. This dataset includes 85 template structures and three subsets of 100 predicted structural models based on these template structures for which the interactions are ranked as high, low and very low quality referring to the structures having the highest score, 50% probability, and 25% probability of existence, respectively. First, the consistency of the predictions has been tested between the template and structural models, and then the interaction type based on the dynamics has been assigned. The dataset is given in S2 Table.

### Gaussian Network Model

GNM [44, 45] is the simplest elastic network model at residue level, where residue pairs with their alpha carbons located within a cut-off radius (r_c_), are assumed to be connected by harmonic springs. The potential function for a protein structure of N residues in the elastic network description is given as

$${\text{V}}_{\text{GNM}}=\frac{\gamma }{2}\left[\sum_{\text{i},\text{j}}^{\text{N}}\Gamma_{\text{ij}}{\left(\Delta R_{\text{i}}-\Delta R_{\text{j}}\right)}^{2}\right]$$ (1)

where γ is the spring constant and **ΔR** refers to the fluctuation **R** vector of each residue at its alpha carbon position. **Γ** is the Kirchhoff matrix defined as

$$\Gamma =\left\{ \begin{array}{l} -1\;if\;i\neq j\;and\;{\text{R}}_{\text{ij}}\leq {\text{r}}_{\text{c}}\\ 0\;if\;i\neq j\;and\;{\text{R}}_{\text{ij}}>{\text{r}}_{\text{c}}\\ -\sum_{i,j\neq i}\Gamma_{\text{ij}}\;if\;i=j \end{array}\right.$$ (2)

R_ij_ is the distance between alpha carbon atoms i and j. r_c_ is taken as 7 Å. The diagonal Lambda_ii term is the degree of a node and a measure of the local packing density around a given residue.

The correlation between equilibrium position fluctuations, **ΔR**_**i**_ and **ΔR**_**j**_, of residues i and j forms the covariance matrix given as

$$\langle \Delta R_{\text{i}}\cdot \Delta R_{\text{j}}\rangle =\left(\frac{3{\text{k}}_{\text{B}}\text{T}}{\gamma }\right){\left[\Gamma^{-1}\right]}_{\text{ij}}=\left(\frac{3{\text{k}}_{\text{B}}\text{T}}{\gamma }\right){\left[U\Lambda^{-1}U^{\text{T}}\right]}_{\text{ij}}=\left(\frac{3{\text{k}}_{\text{B}}\text{T}}{\gamma }\right)\sum_{\text{k}}{\left[\lambda_{\text{k}}^{-1}u_{\text{k}}u_{\text{k}}^{\text{T}}\right]}_{\text{ij}}$$ (3)

where **U** is an orthogonal matrix whose columns **u**_**i**_ are the eigenvectors of the Kirchhoff matrix and **Λ** is a diagonal matrix whose elements λ_i_ represent the eigenvalues, k_B_ is the Boltzmann constant, and T is the absolute temperature in Eq 3. The slow modes with lower eigenvalues contribute to global cooperative motions, while the fast modes with higher eigenvalues describe local fluctuations. The normalized values of the correlation between residue fluctuations range between +1 and -1.

#### Motion and interaction

The covariance matrix (Eq 2) of a mode of motion divides the structure into dynamic domains: clusters of amino acids that are in close contact with each other and that move collectively in one direction [48]. The motion could be along the eigenvector, marked by correlation of +1, or in opposite direction, with correlation of –1. The sign changes mark the positions of the hinges at the interface between the dynamic domains. When two or more modes are superimposed, the fluctuations along the two eigenvectors are superimposed and the correlations can vary in the range +1 to -1, reflecting the average behavior. For example, the average of the two slowest modes amalgamates the two most global conformational changes accessible to a given complex structure. Incorporation of higher modes integrates other global, and subsequently also local, motions.

The conformational transitions of proteins are intimately related to their function. With the premise of the link between the dynamic infrastructure provided by the interacting chains and the interface type, we propose a dynamic measure based on GNM through the analysis of obligatory versus non-obligatory complex structures in the dataset: The dynamics are often dominated by the two slowest modes, yet further refined by the next slow modes; dynamic domains capture the global connectivity similarity for obligatory versus non-obligatory complex structures.

### Server

Given a protein complex structure, the DynaFace web-server builds the covariance matrix and uses it to classify the inter-protein interfaces as obligatory vs. non-obligatory. Various combinations of different sets of modes are used in order to best exploit the dynamic correlation patterns. For the most part, the two slowest modes capture the pattern of the dynamic domains with respect to the interaction type. The ten slowest modes contribution is significant, but higher modes lead to fine-tuning. As a result, DynaFace uses the ten slowest modes (+ all modes) to increase the prediction accuracy. The addition of the all-modes terms increase the performance in comparison to using only the slowest 10 modes, as well as in comparison to using the 10 slowest plus modes 11 and higher.

The underlying scoring function of DynaFace basically reflects the pattern of the cross-correlation map. The following seven attributes are calculated: The average of all (A), negative (N) and positive (P) correlations between residue fluctuations of two interacting subunits in the average ten slowest (s) and all (a) modes of motion, and the number of associating regions between two subunits in all modes (AR), defined in the following paragraph. For each attribute, a knowledge-based decision parameter (threshold) is set based on the corresponding correlation values of 139 obligatory and 246 non-obligatory complex structures in the whole dataset; see S3 Table for the individual performance of each attribute. The *subunit* refers to the interacting partner unit; a single chain or more than one chain. A decision outcome D is calculated with respect to an obligatory interaction as follows

$$\text{D}=\left({\text{A}}_{\text{a}}>{\hat{\text{A}}}_{\text{a}}\right)+\left({\text{N}}_{\text{a}}>{\hat{\text{N}}}_{\text{a}}\right)+\left({\text{P}}_{\text{a}}<{\hat{\text{P}}}_{\text{a}}\right)+\left({\text{A}}_{\text{s}}>{\hat{\text{A}}}_{\text{s}}\right)+\left({\text{N}}_{\text{s}}>{\hat{\text{N}}}_{\text{s}}\right)+\left({\text{P}}_{\text{s}}<{\hat{\text{P}}}_{\text{s}}\right)+\left({\text{AR}}_{\text{a}}>\hat{{\text{AR}}_{\text{a}}}\right)$$ (4)

Where, the hat over each attribute refers to a pre-determined threshold value. If the majority, i.e., four or more of the seven binary attributes satisfy the criteria for the obligatory interaction (D ≥ 4), the decision is *obligatory*; otherwise it is *non-obligatory*. The threshold value for each attribute is given in S4 Table. Note that lower P with higher A and N values in an obligatory interfaces is the result of the manifestation of larger number of weak to strong positively correlated and weak negatively correlated residue pairs A large value of AR means a large number of associating regions between two subunits, which is an indicator of obligatory interface.

Associating regions (AR, the last attribute in Eq 4) refer to the interface residues that transduce the motion between the two subunits. Residues that correlate dynamically more strongly to the juxtaposed subunit than to their own subunit are defined as anchors. AR is the ratio of the total number of anchor residues from both chains to the sum of amino acids in the whole complex. Anchor residues are more abundant in obligatory compared to non-obligatory complexes.

Two different sets of thresholds are used depending on complex size: One set when both subunits are >65 residues, and another when the complex includes a subunit of less than 65 residues. The threshold values were also tested over sub-datasets produced by randomly dividing the original dataset into five groups.

In test trials of DynaFace using only the two and only the ten slowest modes, three attributes corresponding to A, N and P were calculated. If two out of three decision variables satisfy the criteria for obligatory interaction in these cases, the decision outcome D is considered *obligatory* otherwise *non-obligatory*. The resulting predictions are presented in *Results and Discussion*.

**Fig 1:**
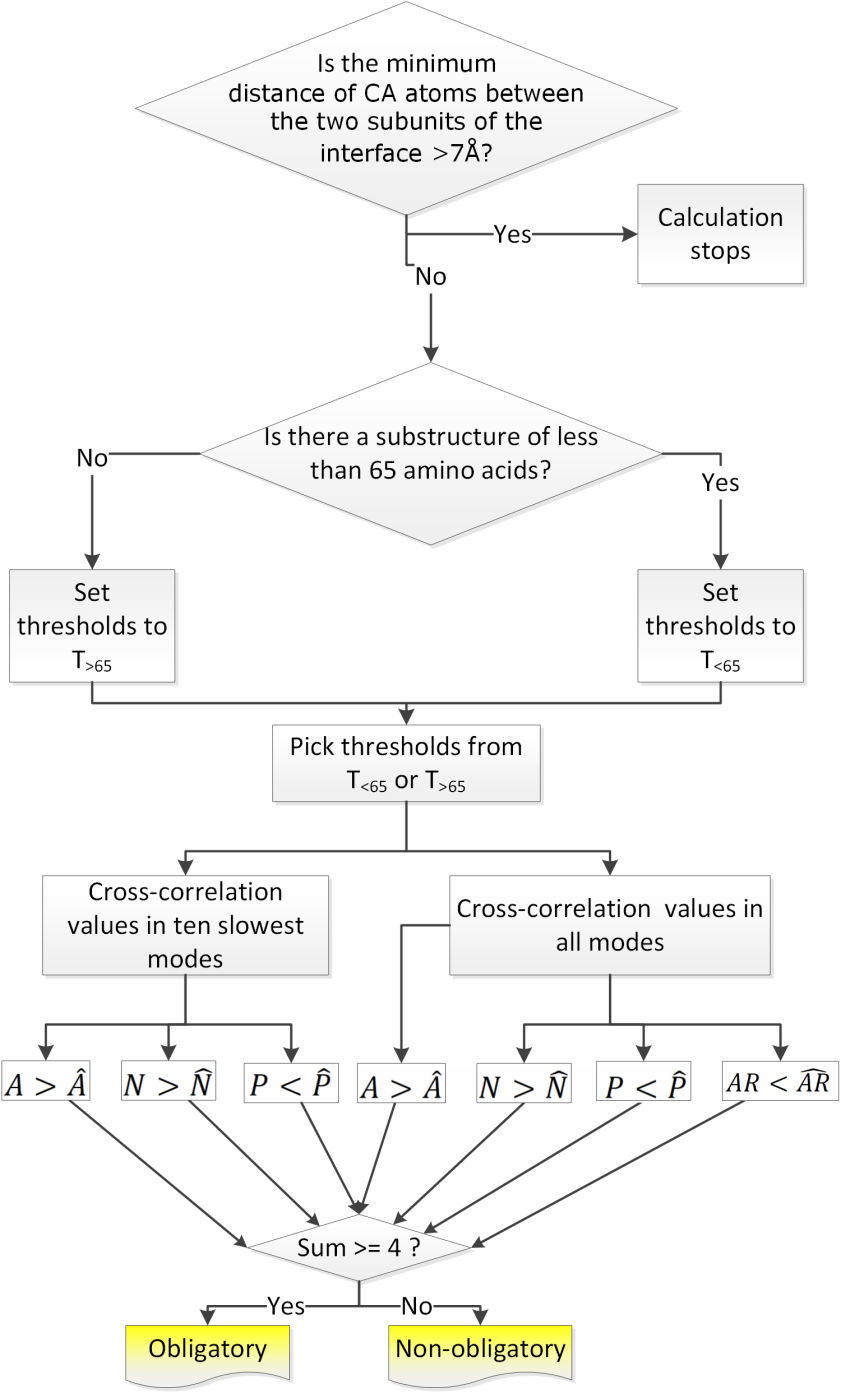
Flowchart of the DynaFace web server. A is the average of all cross-correlation values between different subunits, N is the average of negative cross-correlation values between different subunits, P is the average of positive cross-correlation values between different subunits, AR is the ratio of associating regions in the protein, and superscript ^ stands for the pre-determined threshold for that parameter.

A more formal description of the calculations is provided in *Supplementary Data*. The flow chart of the algorithm is given in Fig 1. The confidence levels for obligatory and non-obligatory complex structures in the dataset are given in S1 Fig.

The DynaFace input can be a PDB ID or an uploaded coordinate file in standard PDB format. The output includes: predicted interface type, dynamic structural domains, cross-correlation maps and their projections on the structure’s ribbon diagram. In the case of non-obligatory interactions, anchors are listed with anchoring groove residues, which are the residues in the juxtaposed subunit that display the highest correlations (top three) in all modes with the anchor residue across the interface.

## Results and Discussion

The PDB includes many interfaces, some obligatory, some non-obligatory, and some artifacts due to crystal-packing. There are methods to filter out crystal-packing interfaces, which work at least to some extent. We offer a complementary method that can differentiate between obligatory and non-obligatory interfaces based on the dynamics of the structural complex. For completeness, and because some crystal-packing artifacts might ‘sneak in’, we also check whether they confuse our method. The results show that DynaFace might mistakenly assign crystal-packing artifacts to be non-obligatory interfaces. We also use our methodology on a dataset of predicted complex structures as well as their corresponding template structures [6] to suggest the interaction type for each.

### Motion and interaction: Global dynamics

Equilibrium residue fluctuations define dynamic domains, where the cooperative modes of motion are the main determinants. The collective dynamics facilitates biological function(s), and correlated motions within the structure could reveal functional predispositions. Thus, the expression of the dynamic couplings across the inter-subunit interface could reveal whether the complex is obligatory or non-obligatory. To examine this we start with two exemplary dimeric complexes. The first is the obligatory homodimer 1QU7 (Cytoplasmic domain of a serine chemotaxis receptor) [49], and the second is the non-obligatory heterodimer 2SIC (SubtilisinBPN' in complex with Streptomyces subtilisin inhibitor) [50].

**Fig 2:**
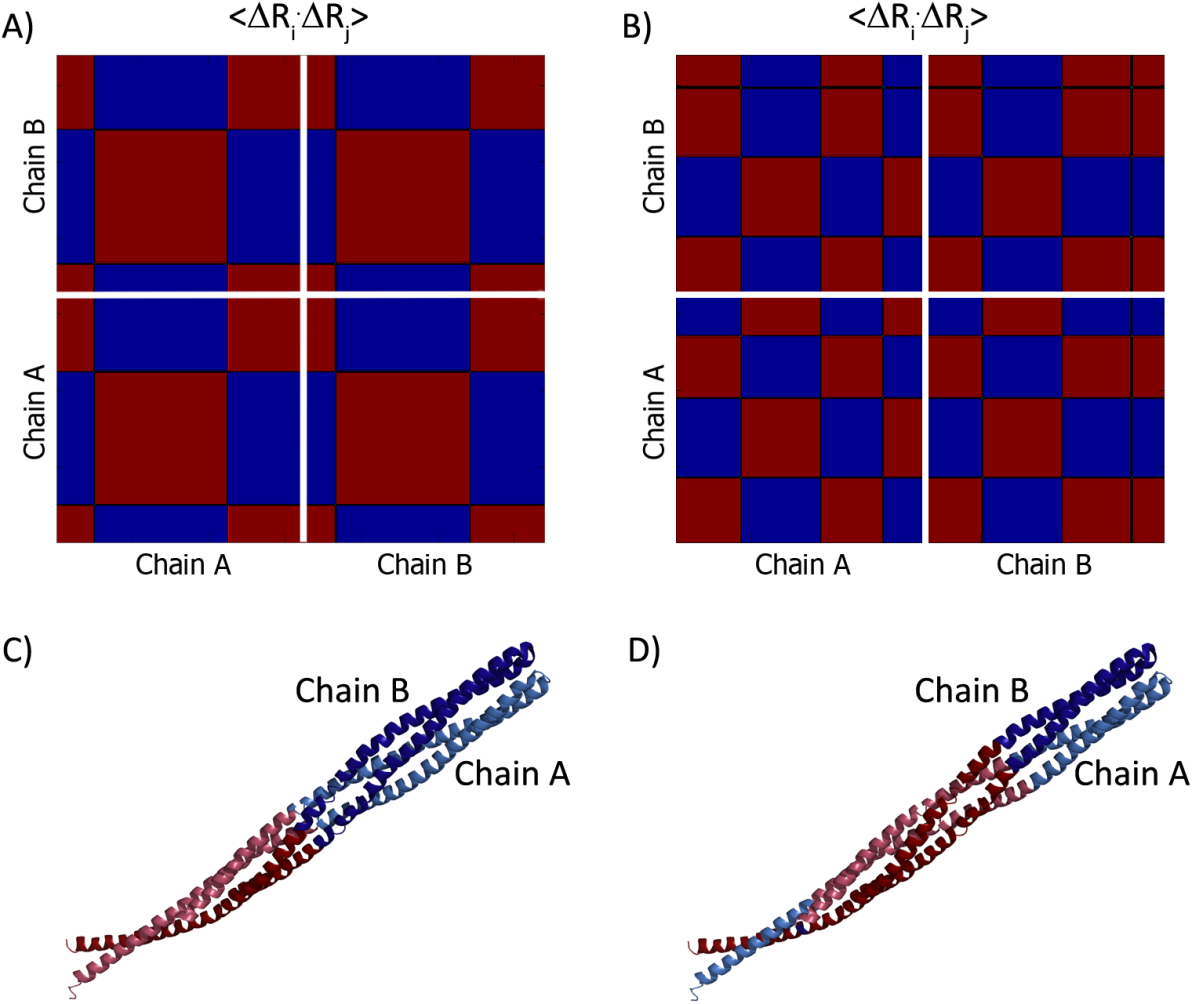
Dynamics of an obligatory complex. Slow modes in the cytoplasmic domain of the homodimeric serine chemotaxis receptor (1QU7 [49]). The upper panels show the matrices of correlations between residue fluctuations, 〈Δ**R**_i_ ⋅ Δ**R**_j_〉, of mode 1 (A) and mode 2 (B), with negative and positive marked as blue and red, respectively. The boundaries between the subunits are marked in white. The lower panels show projection of the correlations on the 3D-structure: C- mode 1 and D- mode 2. The subunits are shown on the PyMOL [61] figures in lighter and darker versions of the same colors.

**Fig 3:**
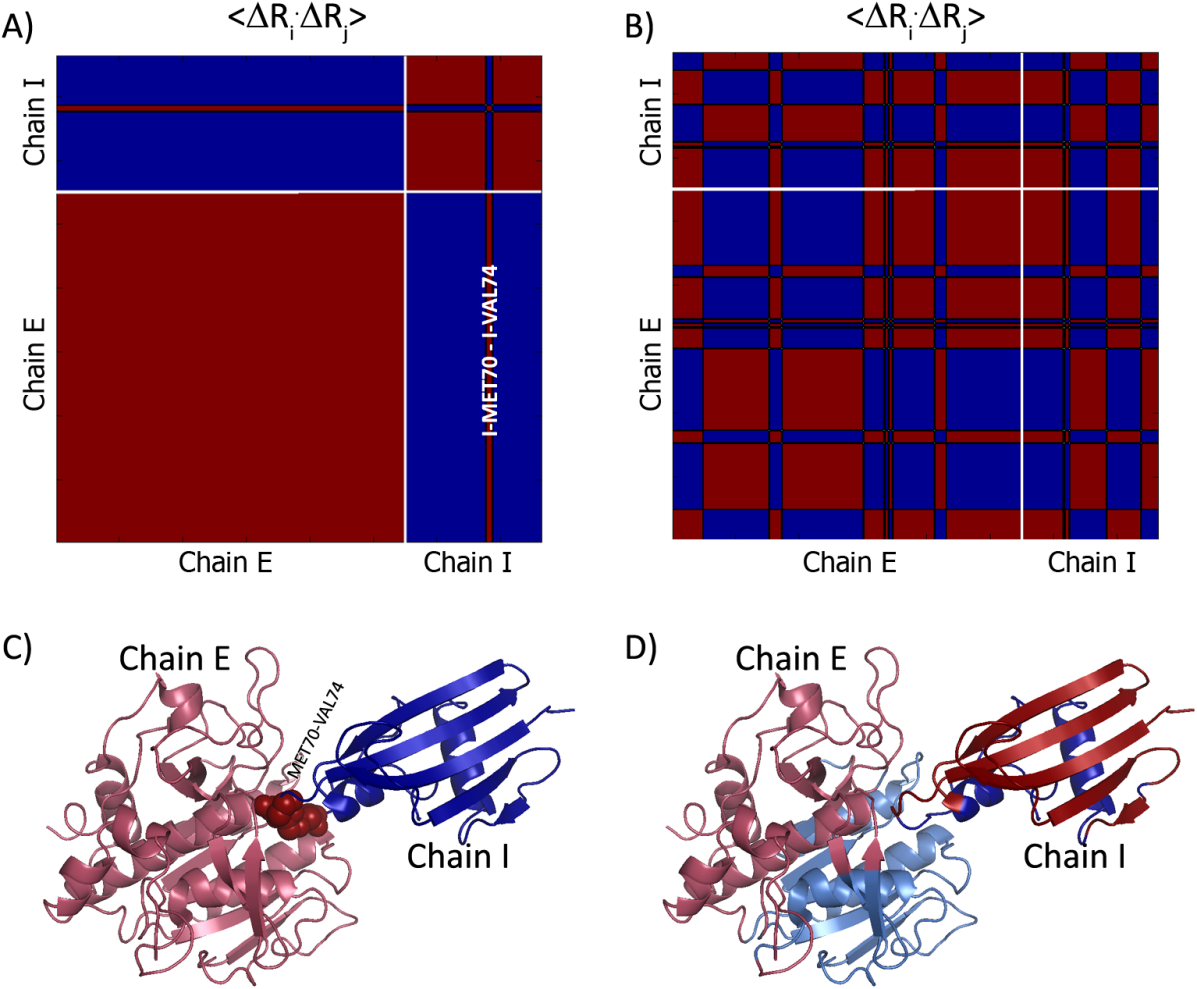
Dynamics of a non-obligatory complex. Slow modes of motion in the complex between subtilisin BPN’ and proteinaceous inhibitor from Streptomyces (2SIC [52]). The upper panels show the matrices of correlations between residue fluctuations, 〈Δ**R**_i_ ⋅ Δ**R**_j_〉, of mode 1 (A) and mode 2 (B) with negative and positive marked as blue and red, respectively. The boundaries between the subunits are marked in white. The lower panels show projection of the correlations on the 3D-structure: C- mode 1 and D- mode 2. The subunits are shown on the PyMOL [61] figures in lighter and darker versions of the same colors.

Cross-correlations of residue fluctuations in the two slowest modes for these dimers are presented in Fig 2 and Fig 3, respectively. Reassuringly, the positioning of the dynamic domains relative to the inter-subunits interfaces differs between the obligatory (Fig 2) and non-obligatory (Fig 3) interactions. The obligatory complex features mostly dynamic domains that share segments between the two subunits, as in the two slowest modes (Fig 2); indeed, the complex’s dynamics is dominated by such modes (S2A Fig, S2B Fig and S2C Fig). The dynamic domains of some of the modes of motion of the non-obligatory complex also share segments across the subunits interface (e.g., the second slowest mode, Fig 3). However, the complex’s dynamics is dominated by modes with correlations only within the individual subunits, such as the slowest mode (Fig 3C, S2D Fig, S2E Fig and S2F Fig). Interestingly, the slowest mode of the non-obligatory complex also manifests ‘anchor’ behavior, where an anchor in the inhibitor (residues MET70-VAL74) is dynamically correlated with the enzyme. The anchor MET70-VAL74 correlates with the majority of the enzyme residues in the average over the two slowest modes (S2D Fig). A subset of residues (GLY100, SER125, THR220) correlate with the anchor also in the average over all modes. These are referred to as ‘anchoring groove’ residues (S2F Fig). This behavior is typical for non-obligatory complexes; the anchors are lesser in number compared to obligatory complexes but provide a means for transduction between the intact chains. This has led us to add the last term in the formula used to discriminate between the interface types (Eq 4). More detailed description of the dynamics of the two complexes is provided in the following two sections.

#### Obligatory interaction: The cytoplasmic domain of a serine chemotaxis receptor (1QU7)

The cytoplasmic domain of the serine chemotaxis receptor of Escherichia coli, is a homodimeric complex with two protein chains of 227 amino acids, marked as A and B [49]. Each subunit is an elongated helical hairpin and the subunits intertwine to form a four helix bundle. In contrast to many other receptors, this receptor is dimeric regardless of ligand binding, and its signaling is independent of the monomer-dimer equilibrium [51].

According to the slowest mode, the dimeric structure has four hinges, two in each subunit; A: HIS328/LEU329, A: THR450/ARG451, B: HIS328/LEU329, and B: ARG451/VAL452 (Fig 2A, S5 Table). The hinges align along a plane that passes through the geometrical center of the complex. The hinge plan separates the dimer into two dynamic domains, each of which includes segments from the two subunits (S5 Table). The second slowest mode describes another type of motion where the underlying dynamic domains (S5 Table) are coordinated by hinge residues ASP353/ILE354, GLY426/LYS427, VAL483/THR484 in chain A, and ASP353/ILE354, GLY426/LYS427 in chain B. These dynamic domains also mix segments from both subunits. The slowest mode dominates the dynamic fluctuations of the dimer. It dominates the average over the two slowest modes (S2A Fig), as well as the averages over the ten slowest (S2B Fig) and all (S2C Fig) modes, which are used to discriminate between interface types. S4 Table shows the threshold values and the attributes of each decision variable for this case as given in Eq 4. The complex satisfies four of seven criteria, and is classified as obligatory, as it should.

#### Non-obligatory interaction: Subtilisin BPN' in complex with streptomyces subtilisin inhibitor (2SIC)

The complex between subtilisin BPN' (chain E, residues 1–275) with its streptomyces inhibitor (chain I, residues 7–113) is an example for non-obligatory interaction (PDB ID: 2SIC; [52]). The slowest mode of motion divides the structure into two dynamic domains with a hinge plane at the enzyme-inhibitor interface (Fig 3A and 3C, S6 Table). It is noteworthy that here each subunit is, in essence, an autonomous dynamic domain, which is often the case for non-obligatory interactions. The slowest mode also predisposes an anchor (MET70-VAL74) for an anchor-and-groove behavior, which is a hallmark for non-obligatory interactions.

The second slowest mode with hinge sites at SER24/ASN25, VAL150/ALA151 of the enzyme (chain E) and HIS43/PRO44, PRO72/MET73, ASN99/GLU100 of the inhibitor (chain I) also separates the structure into two dynamic domains (Fig 3B and 3D, S6 Table). This mode resembles modes that are observed in obligatory complexes in that the dynamic domains mix segments from both subunits.

The two slowest modes dominate the average dynamic behavior of all modes (S2D–S2F Fig), which displays that the dynamic linkage between the two proteins is mostly through a groove, comprising residues GLY100, SER125 and THR220 of the enzyme (chain E), surrounding an anchor, comprising residues MET70-VAL74 of the inhibitor (chain I, S2F Fig). The residues of the anchor are dynamically coupled more strongly to the amino acids of the enzyme than the amino acids of their own chain, i.e., the inhibitor. The anchor-and-groove maintains the dynamic coupling between the subunits, presumably also the functional interaction. S4 Table shows the threshold values and the attributes of each decision variable for this case as given in Eq 4. Four of the seven attributes (AR, A_a_, N_a_, A_s_) suggest that this protein is non-obligatory, as it really is.

### Motion and interaction: Large-scale evaluation

The examples above suggest that it could be possible to discriminate between obligatory (1QU7) and non-obligatory (2SIC) interactions based on their dynamic footprints (Fig 2 and Fig 3). To examine how feasible it is we conduct GNM calculations of the 139 obligatory and 246 non-obligatory protein complexes in our dataset and analyze dynamic correlation maps as described in the flow chart of Fig 1. Preliminary tests are conducted using the fluctuations of the two slowest, ten slowest, and all modes of motion. The sense and magnitude of the correlations between intra- and inter-chain residue fluctuations and the number of anchor regions are analyzed for a best scoring metric to predict the interface type.

The results, summarized in Table 1, show that even estimates based only on the two slowest modes yield a significant success rate for the prediction: 62.6% (87 out of 139) and 64.6% (159 out of 246) for obligatory and non-obligatory interfaces, respectively. The success rates increase to 69.1% (96 out of 139) and 72.8% (179 out of 246), respectively, when the ten slowest modes are considered. Furthermore, combining the ten slowest and all modes, the success rates are as high as 84.2% (117 out of 139) and 82.5% (203 out of 246), respectively. To examine the stability of the results obtained with the latter setting we divide the dataset randomly into five subsets. Reassuringly, the success rates of the five sets are 84.6 ± 5.8% and 82.6 ± 4.6% for obligatory and non-obligatory complexes, respectively. With the latter results, the server now considers the ten slowest and all modes of motion in the background analysis for the best decisive outcome. These results suggest that the dynamics is able to capture largely the global topological similarity of obligatory and non-obligatory complex structures and provides a plausible measure for the interface type prediction. We should keep in mind, though, that there is continuous spectrum of dynamic behaviors from non-obligatory to obligatory interactions, rather than a clear separation into two clusters. Our choice of thresholds is optimal, albeit arbitrary.

**Table 1:** The success rate of DynaFace.

	Obligatory Interfaces	Non-obligatory Interfaces	Overall
Two slowest modes	62.6%	64.6%	63.9%
Ten slowest modes	69.1%	72.8%	71.4%
Ten slowest modes combined with all modes	84.2%	82.5%	83.1%

The success rate of DynaFace to reproduce the interface annotations of S1 Table based on various normal mode combinations.

As a reference we examine the success rates of NOXclass [38] on the same dataset (Table 2). NOXclass fails to process the query for PDB entries of more than two chains. Thus, only the binary complexes in the original set are used. The success rates are 74.2% (89 of 120) for obligatory complexes, and 66.5% (107 of 161) for non-obligatory, respectively. DynaFace’s success rates on the same set are significantly higher: 81.7% (98 out of 120 cases) and 83.2% (134 out of 161 cases), respectively. It is noteworthy that the two approaches are complementary to each other in that they are based on orthogonal properties; NOXclass uses various local sequence- and structure-based properties, and DynaFace is based on the fluctuation dynamics as a single and mainly global property. Thus, it could be possible to combine DynaFace and NOXclass to improve the performance further.

**Table 2:** The success rate of NOXclass and DynaFace for the binary complexes in the set of S1 Table.

	Obligatory Interfaces	Non-obligatory Interfaces	Overall
NOXclass	74.2%	66.5%	69.8%
Dynaface	81.7%	83.2%	82.6%

A similar comparison has been made with PiType. PiType, which uses Uniprot IDs, makes use of already existing interaction networks in its database, and most of the cases in our dataset are not included in their database. We thus made a partial comparison on 62 proteins (45 non-obligatory, 17 obligatory) that exist both in our dataset and PiType’s. DynaFace successfully reproduced annotation of 85.4% (82.3% of obligatory and 86.7% of non-obligatory complexes) of the complexes, while PiType reproduced only 80.6% (70.6% of obligatory and 84.4% of non-obligatory complexes) of these complexes, respectively.

### DynaFace classifies crystal packing as non-obligatory interfaces

Due to the close packing of protein molecules in crystals, some interactions observed in X-ray crystal structures are non-biological. PDB entries are thus known to contain artifacts of crystallization that do not have any biological relevance [10]. It is of interest to examine how these are classified by DynaFace.

Because a reliable gold-standard of non-biological interfaces is not available, we compiled one based on previous studies. The starting point was a dataset of 63 large crystal packing interfaces with buried surface area of 800 Å^2^ or more, where the monomeric state was confirmed by biochemical or biophysical studies [53]. DynaFace calculations failed for four of the complexes due to the appearance of a singular connectivity matrix due to the topology of the complex structure where the minimum distance of alpha carbon atoms between the two subunits of the interface is > 7 Å. These complexes have been removed from the dataset. Of the rest of the 59, three were classified as obligatory and the rest as non-obligatory. Next, we applied existing computational tools for the detection of crystal packing interfaces; PQS [54], PITA [55], PISA [56] and PInS [57] and NOXclass [38]. The DIMOVO tool [58] was excluded because it was trained on the present dataset. Table 3 summarizes the success rate of each server and S7 Table shows the detailed results. Each and every method classifies many interfaces as biologically meaningful, rather than being crystal artefacts, suggesting that the methods or dataset are imperfect.

**Table 3:** The success rate of PQS, PINS, NOXclass, PISA and DynaFace in the detection of the crystal packing interfaces of S7 Table.

PQS	PINS	NOXclass	PISA	DynaFace
61.9%	53.9%	76.2%	55.5%	88.9%

To be on the safe side, we filtered the dataset and applied a particularly restrictive criterion, keeping only 13 crystal-packing interfaces with consensus prediction (Highlighted structures in S7 Table). DynaFace classifies all the remaining interfaces as non-obligatory. Interestingly, ten of them do not display anchors, the key indication of non-obligatory interfaces. The outliers are 1a7v, 2atj and 3ng1, which display anchoring behavior between subunits. The dynamic behavior of crystal and biological non-obligatory complex structures needs to be further investigated on a larger clean dataset of crystal complex structures.

### Prediction with models of protein complexes (PrePPI)

Having validated DynaFace on documented cases, we used it also to analyze interfaces of 300 model structures, predicted by the PrePPI tool [6]. The structural models were constructed using a combination of structural and non-structural interaction clues and assigned confidence levels “high”, “low” or “very low”. For completion we also analyzed the 85 real dimeric structures used as the templates for the models, assuming that agreement between the classification of the interface of the model and template is indicative of the prediction accuracy. The results are provided in S2 Table. Reassuringly, the agreement between DynaFace predictions of model and template correlated with the confidence of the model according to the PrePPI estimate: 86.0% agreement for the 100 models of the ‘high’ confidence, 72.6% for the 100 with ‘low’ confidence, and 57.0% for the 100 with “very low” confidence.

Among 100 model structures in the ‘high’ PrePPI category, 93 are predicted to be obligatory. Of the templates of these, 86 are also predicted to be obligatory. Only 7 of the model structures in the ‘high’ category are predicted to be non-obligatory, but their templates are not. Of the 100 PrePPI dimers in the “low” category, 22 are predicted to be obligatory, 73 to be non-obligatory, and 5 could not be processed because the subunits were over 7 Å apart from each other in the model. Consensus between DynaFace prediction for model and template is obtained for only one of the 22 obligatory dimers but for 68 of the 73 non-obligatory interfaces. Of the 100 PrePPI structures in the “very low” category, 27 and 73 structures are predicted respectively to be obligatory and non-obligatory; consensus with the prediction for the template is obtained for 10 of the 27 obligatory interfaces and 47 of the 73 non-obligatory ones.

In total there are 76 cases of disagreement in the prediction of the interface type between the model and template. Examination showed that in 52 cases the disagreement is related to large size differences between the polypeptide chains. That is, when a short chain in the template corresponds to a much larger chain in the model or vice versa. The size difference pronouncedly affects the global dynamics, which has significant contribution here to classify the interface type. As an example, the 8API template, predicted as non-obligatory, was used to model P29508_P01009, P35237_P01009, P48594_P01009, P50453_P01009, Q86WD7_P01009, where a short template chain of 36 amino acids corresponds to 369 in the model, which is predicted to be obligatory. We could not find an obvious reason for the observed disagreement in the rest of the cases (24, i.e., 8%) and we attribute it to parametric error in the twilight zone between obligatory and non-obligatory interfaces.

### A case study with a modeled complex structure

The NMR structure of the transforming growth factor beta 1 (TGF-B1; 1KLD [59]) was used as a template for PrePPI modeling of ten dimer structures in the “high” confidence category and one in the “very low” category. TGF-B1 is a multifunctional cytokine with stimulatory and inhibitory effects. Its mature form is homodimeric. Indeed, the template is predicted to be obligatory as it should [59]), and so are all the PrePPI models assigned with high confidence level. In contrast, the one PrePPI model, assigned with very low confidence is predicted to be non-obligatory.

As a sample case, the underlying dynamics for the structural model P09529_P01137 is presented in S4 Fig and S8 Table. The RMSD between one of the structural models P09529_P01137 in the “high” category that was analyzed here and the template structure, chains A and B of 1KLD, is 1.6 Å. Both of these structures are found to be obligatory, in line with the experimental findings of this protein structure [60]. According to the slowest mode, the homodimeric structure has five hinges; three in chain A: GLU333/GLY334, CYS372/ILE373 and CYS404/GLY405, and two in chain B: GLY46/PRO47 and CYS77/CYS78. The hinges of both chains align to a single plane, dividing the structure into two dynamic domains. In the second slowest mode, the dynamic domains are defined by hinge residues PHE309/ILE310, TYR327/TYR328, THR379/MET380, ASP395/VAL396 in chain A and LEU20/TYR21, ALA41/ASN42, GLU84/PRO85, MET104/ILE105 in chain B. The two slowest modes dominate the overall dynamic behavior in this case. Here, the intra- and inter-chain nature of the cooperativity is indistinguishable, assumed hallmark of obligatory interaction.

### Conclusion

Protein-protein complexes are involved, in essence, in all intra- and inter-cellular processes, and the nature of the inter-protein interface determines mechanistic aspects of the interaction. The interfaces have been evolutionarily-designed to enable transduction between the proteins, suggesting that analysis of the dynamics of the complex can reveal the nature of the interaction. The global dynamics of protein complexes should be particularly crucial for function. Following this hypothesis, DynaFace exploits the dynamics of protein complexes in order to detect obligatory vs. non-obligatory interactions among the subunits. The global perspective of interactions across the subunits interface is described mainly by the dynamics of the structural complex, which is not easily accessible by studying only local sequence and structural properties. The dynamic domains, the motions of sub-structural units and how they cooperate with respect to the interacting chains, i.e. the dynamic infrastructure provided by the interacting chains, overall captures the global connectivity similarity for the obligatory and non-obligatory complex structures. To this end, the dynamics of protein complexes in terms of the motions of their dynamic domains and how they are dynamically coupled for their function is of importance for design and function modification of proteins. It is important to note that overall, the interaction spectrum is continuous and does not readily land itself to any discrete classifications. Thus, it is not surprising that DynaFace predictions are imperfect.

DynaFace could readily be embedded in other tools for predicting interface type. Such tools often use local characteristics, which are complementary to the global features used in DynaFace. Indeed, an approach that combines both local and global features would provide a means to discover further and more novel aspects of biological processes.

## Supporting Information

S1 Fig Obligatory and non-obligatory interactions often differ in their dynamic characteristics. The results obtained using the dataset of S1 Table, which includes a total of 139 obligatory and 246 non-obligatory complexes. The X-axis represents the value of the decision outcome D in Eq 4, ranging from 0 to 7; the Y-axis shows the fraction of the protein complexes having that decision outcome value D. In DynaFace, complexes assigned a D value greater than or equal to 4 is predicted to be obligatory; otherwise non-obligatory.
(TIF)

S2 Fig The correlation between residue fluctuations of an obligatory vs. non-obligatory complex. The upper panels show the correlations 〈**ΔR**_**i**_ ⋅ **ΔR**_**j**_〉 for the average over the two slowest modes (A), the ten slow modes (B), and all modes (C) of motion for an obligatory interface, 1QU7 [49]. The lower panels (D, E, and F) show the respective correlations for a non-obligatory interface, 2SIC [52].
(TIF)

S3 Fig The non-obligatory interaction between the enzyme and inhibitor in the 2SIC complex is mediated using anchor and groove. The anchor residues MAT70-VAL74 on the inhibitor (chain I) are shown as solid spheres, and the groove residues GLY100, SER125, THR220 on the enzyme (chain E) are shown as doted spheres. The figure was produced using PyMOL [61].
(TIF)

S4 Fig Slow modes of motion in the structural model P09529_P01137 based on the template NMR structure of the transforming growth factor beta 1 (TGF-B1, PBD ID: 1KLD [59]). The top panels show the matrices of correlations between residue fluctuations, 〈Δ**R**_i_ ⋅ Δ**R**_j_〉, of mode 1 (A) and mode 2 (B) color-coded for negative (blue) and positive (red) correlations. The boundaries between the subunits are marked in white. The bottom panels show projection of the correlations on the 3D-structure: C- mode 1 and D- mode 2. The subunits are shown on the PyMOL [61] figures in lighter and darker versions of the same colors; spheres are the hinge residues.
(TIF)

S1 Table The dataset of 246 non-obligatory and 139 obligatory protein complexes [14, 31]. The original references as well as the references used to update the interaction type are given. DynaFace predictions are also included.
(DOCX)

S2 Table Dataset of 85 template structures and the PrePPI structural models predicted based on these template structures [6] along with their DynaFace predictions. “Model ID”–the PrePPI index number of the predicted dimer model. “Probability”- the PrePPI prediction likelihood: high, low, very low. “Template ID”–the PDB accession number of the template PrePPI used to model the dimer. “Template Chain IDs”–the chains PrePPI used to model the structure. “Model”–DynaFace prediction of the interface type of the model structure: obligatory vs. non-obligatory. “Template”–DynaFace prediction of the interface of the template.
(DOCX)

S3 Table Individual performance of each attribute for obligatory and non-obligatory complex structures in the dataset. (DOCX)

S4 Table The threshold value for the seven dynamic attributes used in a DynaFace calculations. The values of the attributes for two examples are presented, where attributes that exceed the threshold are highlighted in bold. PDB IDs 1QU7 and 2SIC are predicted to be obligatory and non-obligatory, respectively, as they should.
(DOCX)

S5 Table The dynamic building units, structural units and hinge residues of an example obligomer: The homodimeric cytoplasmic domain of the serine chemotaxis receptor (1QU7 [49]). (DOCX)

S6 Table The dynamic building units, structural units and hinge residues of an example non-obligatory dimer: Subtilisin BPN' in complex with its streptomyces subtilisin inhibitor (2SIC [52]). (DOCX)

S7 Table The comparison of the predictions of the servers; PQS, PINS, NOXclass, PISA and DynaFace on the crystal dataset by [53]. (DOCX)

S8 Table The dynamic building units, structural units and hinge residues of the structural model P09529_P01137 based on the template NMR structure TGF-B1 (1KLD[59]). (DOCX)

S1 Method (DOCX)
