# Supplementary figures and images for: DynaFace: Discrimination between Obligatory and Non-obligatory Protein-Protein Interactions Based on the Complex’s Dynamics
Source: PLoS Comput Biol. 2015 Oct 27;11(10):e1004461. doi: 10.1371/journal.pcbi.1004461 (PMC4623975; doi:10.1371/journal.pcbi.1004461)

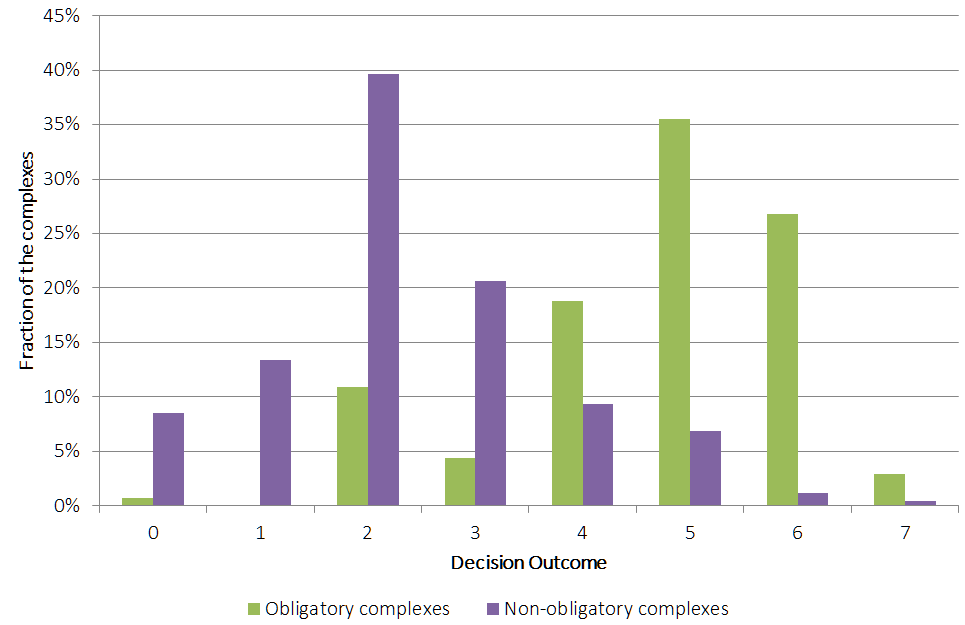

Supplement: S1 Fig — The results obtained using the dataset of S1 Table, which includes a total of 139 obligatory and 246 non-obligatory complexes. The X-axis represents the value of the decision outcome D in Eq 4, ranging from 0 to 7; the Y-axis shows the fraction of the protein complexes having that decision outcome value D. In DynaFace, complexes assigned a D value greater than or equal to 4 is predicted to be obligatory; otherwise non-obligatory. (TIF) [file pcbi.1004461.s001.tif]

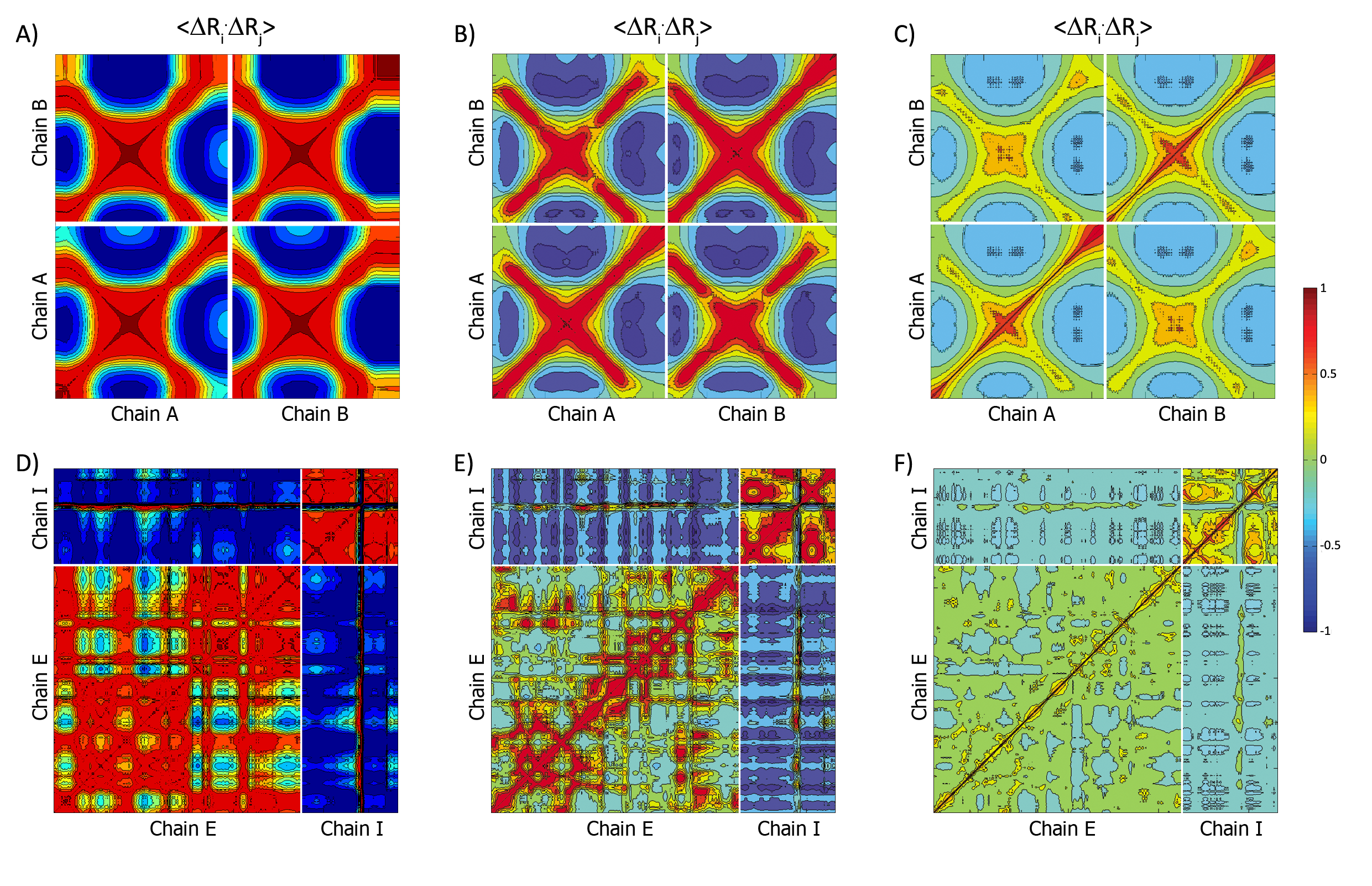

Supplement: S2 Fig — The upper panels show the correlations 〈ΔR i ⋅ ΔR j〉 for the average over the two slowest modes (A), the ten slow modes (B), and all modes (C) of motion for an obligatory interface, 1QU7 [49]. The lower panels (D, E, and F) show the respective correlations for a non-obligatory interface, 2SIC [52]. (TIF) [file pcbi.1004461.s002.tif]

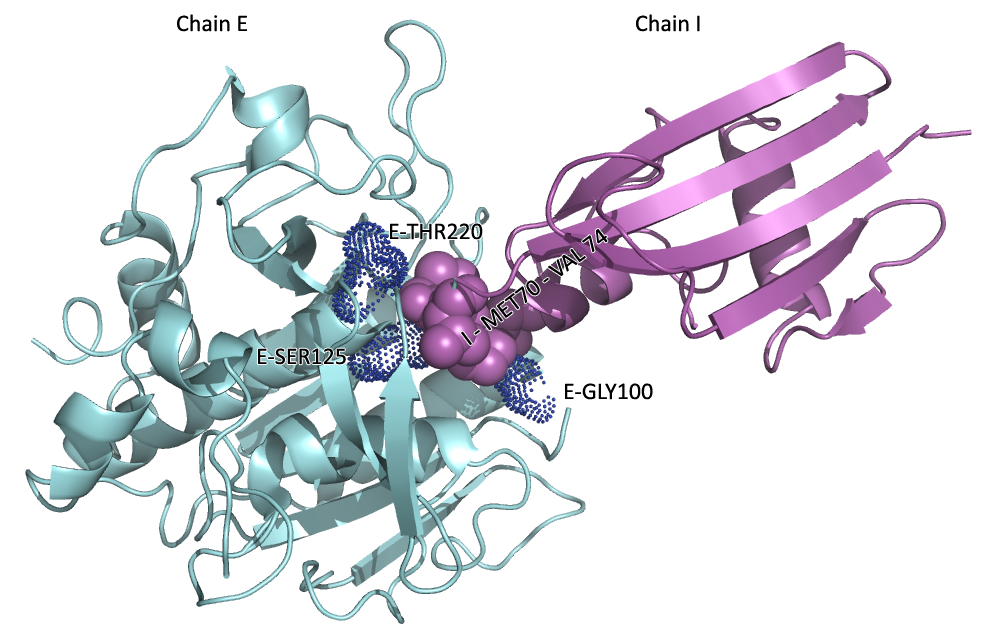

Supplement: S3 Fig — The anchor residues MAT70-VAL74 on the inhibitor (chain I) are shown as solid spheres, and the groove residues GLY100, SER125, THR220 on the enzyme (chain E) are shown as doted spheres. The figure was produced using PyMOL [61]. (TIF) [file pcbi.1004461.s003.tif]

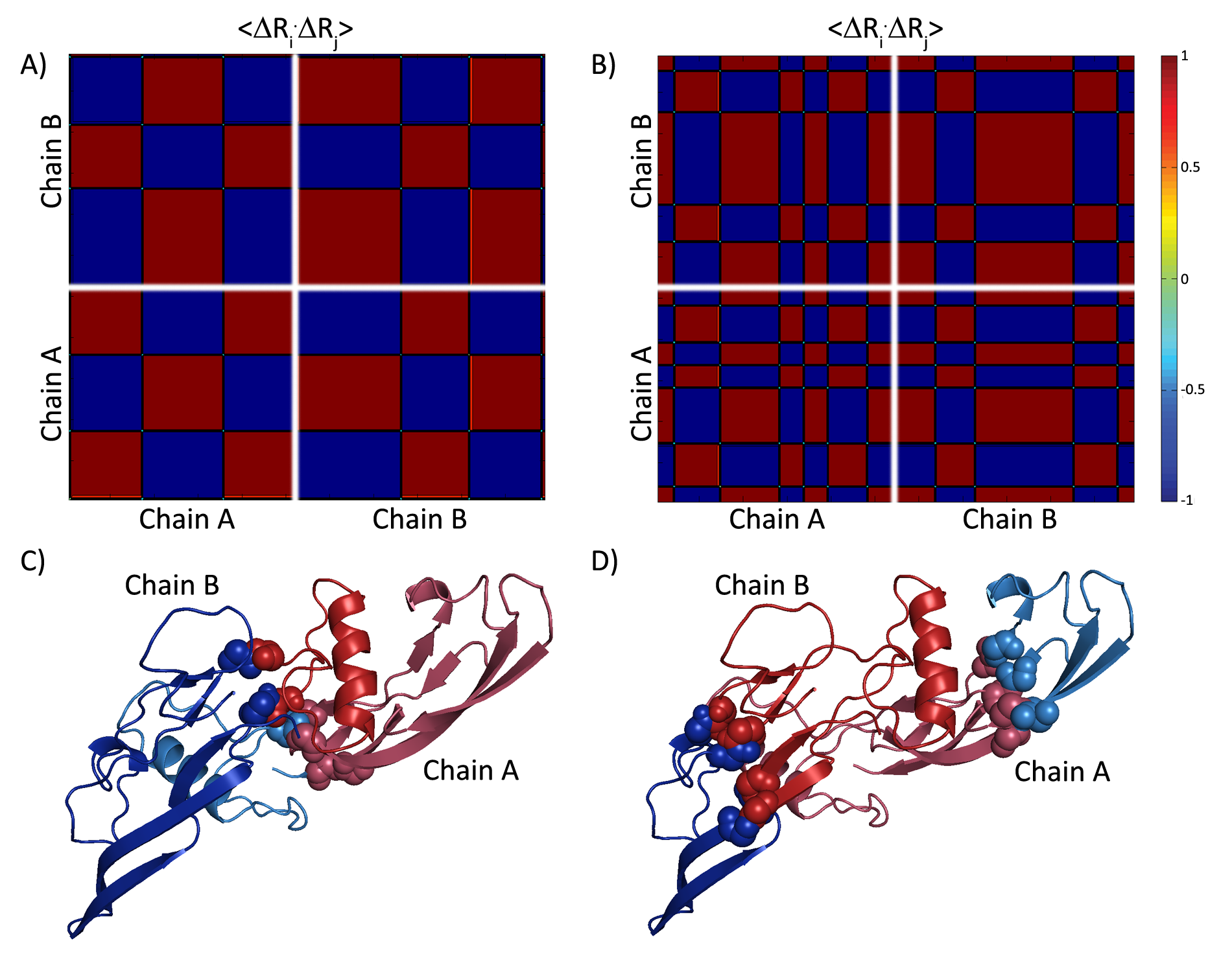

Supplement: S4 Fig — The top panels show the matrices of correlations between residue fluctuations, 〈ΔR i ⋅ ΔR j〉, of mode 1 (A) and mode 2 (B) color-coded for negative (blue) and positive (red) correlations. The boundaries between the subunits are marked in white. The bottom panels show projection of the correlations on the 3D-structure: C- mode 1 and D- mode 2. The subunits are shown on the PyMOL [61] figures in lighter and darker versions of the same colors; spheres are the hinge residues. (TIF) [file pcbi.1004461.s004.tif]
