# Supplementary material for: DynaFace: Discrimination between Obligatory and Non-obligatory Protein-Protein Interactions Based on the Complex’s Dynamics
Source: PLoS Comput Biol. 2015 Oct 27;11(10):e1004461. doi: 10.1371/journal.pcbi.1004461 (PMC4623975; doi:10.1371/journal.pcbi.1004461)
Supplement: S1 Table — The original references as well as the references used to update the interaction type are given. DynaFace predictions are also included. (DOCX) [file pcbi.1004461.s005.docx]

**S1 Table.** **The dataset of 246 non-obligatory and 139 obligatory protein complexes [**[**14**](#_ENREF_14)**,** [**31**](#_ENREF_31)**].**

| **PDB** | **Dataset** | **Res** | **Given dataset type** | **Updated Dataset Type** | **Update Reference** | **Server Result** |
| --- | --- | --- | --- | --- | --- | --- |
| 1a0f.A_B | [[31](#_ENREF_31)] | 2.1 | Obligatory | Obligatory |  | Obligatory |
| 1a14.HL_N | [[14](#_ENREF_14)] | 2.5 | Transient | Transient |  | Transient |
| 1a2k.AB_C | [[14](#_ENREF_14)] | 2.5 | Transient | Transient |  | Transient |
| 1a4i.A_B | [[31](#_ENREF_31)] | 1.5 | Obligatory | Obligatory |  | Obligatory |
| 1a4u.A_B | [[31](#_ENREF_31)] | 1.92 | Obligatory | Obligatory |  | Obligatory |
| 1a4y.A_B | [[31](#_ENREF_31)] | 2 | Transient | Transient |  | Transient |
| 1a6d.A_B | [[14](#_ENREF_14)] | 2.6 | Obligatory | Obligatory |  | Transient |
| 1acb.E_I | [[14](#_ENREF_14)] | 2 | Transient | Transient |  | Transient |
| 1afw.A_B | [[31](#_ENREF_31)] | 1.8 | Obligatory | Obligatory |  | Obligatory |
| 1agr.A_E | [[14](#_ENREF_14)] | 2.8 | Transient | Transient |  | Obligatory |
| 1ahw.AB_C | [[14](#_ENREF_14)] | 3 | Transient | Transient |  | Transient |
| 1aj8.A_B | [[31](#_ENREF_31)] | 1.9 | Obligatory | Obligatory |  | Obligatory |
| 1ajs.A_B | [[31](#_ENREF_31)] | 1.6 | Obligatory | Obligatory |  | Obligatory |
| 1ak4.A_D | [[14](#_ENREF_14)] | 2.36 | Transient | Transient |  | Transient |
| 1akj.AB_DE | [[14](#_ENREF_14)] | 2.65 | Transient | Transient |  | Transient |
| 1ao7.ABC_DE | [[14](#_ENREF_14)] | 2.6 | Transient | Transient |  | Transient |
| 1aom.A_B | [[31](#_ENREF_31)] | 1.8 | Obligatory | Transient | [[62](#_ENREF_62)] | Transient |
| 1aq6.A_B | [[31](#_ENREF_31)] | 1.95 | Obligatory | Obligatory |  | Obligatory |
| 1ar1.AB_CD | [[14](#_ENREF_14)] | 2.7 | Transient | Transient |  | Transient |
| 1aro.P_L | [[14](#_ENREF_14)] | 2.8 | Transient | Transient |  | Obligatory |
| 1at3.A_B | [[31](#_ENREF_31)] | 2.5 | Obligatory | Transient | [[63](#_ENREF_63)] | Transient |
| 1atn.A_D | [[14](#_ENREF_14)] | 2.8 | Transient | Transient |  | Transient |
| 1aui.A_B | [[14](#_ENREF_14)] | 2.1 | Obligatory | Transient | [[64](#_ENREF_64)] | Transient |
| 1ava.A_C | [[14](#_ENREF_14)] | 1.9 | Transient | Transient |  | Obligatory |
| 1avw.A_B | [[31](#_ENREF_31)] | 1.75 | Transient | Transient |  | Transient |
| 1avx.A_B | [[14](#_ENREF_14)] | 1.9 | Transient | Transient |  | Transient |
| 1avz.B_C | [[14](#_ENREF_14)] | 3 | Transient | Transient |  | Transient |
| 1awc.A_B | [[14](#_ENREF_14)] | 2.15 | Transient | Transient |  | Transient |
| 1ay7.A_B | [[14](#_ENREF_14)] | 1.7 | Transient | Transient |  | Transient |
| 1az3.A_B | [[31](#_ENREF_31)] | 2.4 | Obligatory | Transient | [[65](#_ENREF_65)] | Transient |
| 1azz.A_CD | [[14](#_ENREF_14)] | 2.3 | Transient | Transient |  | Obligatory |
| 1b34.A_B | [[31](#_ENREF_31)] | 2.5 | Obligatory | Obligatory |  | Transient |
| 1b3a.A_B | [[31](#_ENREF_31)] | 1.6 | Obligatory | Transient |  | Transient |
| 1b4u.A_B | [[14](#_ENREF_14)] | 2.2 | Obligatory | Obligatory |  | Obligatory |
| 1b5e.A_B | [[31](#_ENREF_31)] | 1.6 | Obligatory | Obligatory |  | Obligatory |
| 1b6c.A_B | [[14](#_ENREF_14)] | 2.6 | Transient | Transient |  | Transient |
| 1b7y.A_B | [[14](#_ENREF_14)] | 2.5 | Obligatory | Obligatory |  | Obligatory |
| 1b8a.A_B | [[31](#_ENREF_31)] | 1.9 | Obligatory | Obligatory |  | Obligatory |
| 1b8j.A_B | [[31](#_ENREF_31)] | 1.9 | Obligatory | Obligatory |  | Obligatory |
| 1b8m.A_B | [[14](#_ENREF_14)] | 2.75 | Obligatory | Obligatory |  | Obligatory |
| 1b9y.AB_C | [[14](#_ENREF_14)] | 3 | Transient | Transient |  | Obligatory |
| 1bbh.A_B | [[31](#_ENREF_31)] | 1.8 | Obligatory | Obligatory |  | Obligatory |
| 1bdj.A_B | [[14](#_ENREF_14)] | 2.68 | Transient | Transient |  | Transient |
| 1be3.CDEGK_A | [[14](#_ENREF_14)] | 3 | Obligatory | Obligatory |  | Obligatory |
| 1bgx.HL_T | [[14](#_ENREF_14)] | 2.3 | Transient | Obligatory | [[39](#_ENREF_39)] | Obligatory |
| 1bi8.A_B | [[14](#_ENREF_14)] | 2.8 | Transient | Transient |  | Transient |
| 1bj1.HL_VW | [[14](#_ENREF_14)] | 2.4 | Transient | Transient |  | Transient |
| 1bjn.A_B | [[31](#_ENREF_31)] | 2.3 | Obligatory | Obligatory |  | Obligatory |
| 1bkd.R_S | [[14](#_ENREF_14)] | 2.8 | Transient | Transient |  | Obligatory |
| 1bml.A_C | [[14](#_ENREF_14)] | 2.9 | Transient | Transient |  | Obligatory |
| 1bo1.A_B | [[31](#_ENREF_31)] | 3 | Obligatory | Obligatory |  | Transient |
| 1bqh.AB_G | [[14](#_ENREF_14)] | 2.8 | Transient | Transient |  | Transient |
| 1buh.A_B | [[14](#_ENREF_14)] | 2.6 | Transient | Transient |  | Transient |
| 1bun.A_B | [[31](#_ENREF_31)] | 2.45 | Obligatory | Transient | [[20](#_ENREF_20)] | Transient |
| 1buv.M_T | [[14](#_ENREF_14)] | 2.75 | Transient | Transient |  | Transient |
| 1bvn.P_T | [[14](#_ENREF_14)] | 2.5 | Transient | Transient |  | Obligatory |
| 1bw0.A_B | [[31](#_ENREF_31)] | 2.5 | Obligatory | Obligatory |  | Obligatory |
| 1byf.A_B | [[31](#_ENREF_31)] | 2 | Obligatory | Transient | [[66](#_ENREF_66)] | Transient |
| 1byk.A_B | [[31](#_ENREF_31)] | 2.5 | Obligatory | Transient | [[67](#_ENREF_67)] | Transient |
| 1bzq.A_L | [[14](#_ENREF_14)] | 2.8 | Transient | Transient |  | Transient |
| 1c1y.A_B | [[14](#_ENREF_14)] | 1.9 | Transient | Transient |  | Transient |
| 1c3o.A_B | [[14](#_ENREF_14)] | 2.1 | Obligatory | Obligatory |  | Obligatory |
| 1c4z.A_D | [[14](#_ENREF_14)] | 2.6 | Transient | Transient |  | Transient |
| 1ccw.A_B | [[14](#_ENREF_14)] | 1.6 | Obligatory | Obligatory |  | Obligatory |
| 1cgi.E_I | [[14](#_ENREF_14)] | 2.3 | Transient | Transient |  | Transient |
| 1clv.I_A | [[14](#_ENREF_14)] | 2 | Transient | Transient |  | Transient |
| 1cmb.A_B | [[31](#_ENREF_31)] | 1.8 | Obligatory | Obligatory |  | Obligatory |
| 1cmx.A_B | [[14](#_ENREF_14)] | 2.25 | Transient | Transient |  | Obligatory |
| 1cnz.A_B | [[31](#_ENREF_31)] | 1.76 | Obligatory | Obligatory |  | Obligatory |
| 1coz.A_B | [[31](#_ENREF_31)] | 2 | Obligatory | Transient | [[68](#_ENREF_68)] | Transient |
| 1cp2.A_B | [[31](#_ENREF_31)] | 1.93 | Obligatory | Obligatory |  | Transient |
| 1cpc.A_B | [[14](#_ENREF_14)] | 1.66 | Obligatory | Transient | [[69](#_ENREF_69)] | Transient |
| 1cs4.AB_C | [[14](#_ENREF_14)] | 2.5 | Transient | Transient |  | Transient |
| 1cse.I_E | [[31](#_ENREF_31)] | 1.2 | Transient | Transient |  | Transient |
| 1cxz.A_B | [[14](#_ENREF_14)] | 2.2 | Transient | Transient |  | Transient |
| 1d2z.A_B | [[14](#_ENREF_14)] | 2 | Transient | Transient |  | Transient |
| 1d4x.A_G | [[14](#_ENREF_14)] | 1.75 | Transient | Transient |  | Obligatory |
| 1d5x.A_C | [[14](#_ENREF_14)] | 2.45 | Transient | Transient |  | Transient |
| 1dce.A_B | [[14](#_ENREF_14)] | 2 | Obligatory | Obligatory |  | Obligatory |
| 1de4.CF_A | [[14](#_ENREF_14)] | 2.8 | Transient | Transient |  | Transient |
| 1dev.A_B | [[14](#_ENREF_14)] | 2.2 | Transient | Transient |  | Transient |
| 1dfj.I_E | [[14](#_ENREF_14)] | 2.5 | Transient | Transient |  | Transient |
| 1dhk.A_B | [[14](#_ENREF_14)] | 1.85 | Transient | Transient |  | Obligatory |
| 1dii.A_C | [[14](#_ENREF_14)] | 2.5 | Obligatory | Obligatory |  | Obligatory |
| 1dj7.A_B | [[31](#_ENREF_31)] | 1.6 | Obligatory | Transient | [[20](#_ENREF_20)] | Transient |
| 1dkf.A_B | [[14](#_ENREF_14)] | 2.5 | Obligatory | Transient | [[39](#_ENREF_39)] | Transient |
| 1dkg.AB_D | [[14](#_ENREF_14)] | 2.8 | Transient | Transient |  | Transient |
| 1dm0.A_BCFDE | [[14](#_ENREF_14)] | 2.5 | Obligatory | Transient | [[39](#_ENREF_39)] | Transient |
| 1doa.A_B | [[14](#_ENREF_14)] | 2.6 | Transient | Transient |  | Transient |
| 1dor.A_B | [[31](#_ENREF_31)] | 2 | Obligatory | Obligatory |  | Obligatory |
| 1dow.A_B | [[31](#_ENREF_31)] | 1.8 | Transient | Transient |  | Transient |
| 1dpj.A_B | [[14](#_ENREF_14)] | 1.8 | Transient | Transient |  | Transient |
| 1dtd.A_B | [[14](#_ENREF_14)] | 1.65 | Transient | Transient |  | Transient |
| 1dtw.A_B | [[14](#_ENREF_14)] | 2.7 | Obligatory | Obligatory |  | Obligatory |
| 1du3.A_DEF | [[14](#_ENREF_14)] | 2.2 | Transient | Transient |  | Obligatory |
| 1dxt.A_B | [[14](#_ENREF_14)] | 1.7 | Obligatory | Obligatory |  | Obligatory |
| 1e0b.A_B | [[31](#_ENREF_31)] | 1.9 | Obligatory | Transient | [[70](#_ENREF_70)] | Transient |
| 1e50.A_B | [[14](#_ENREF_14)] | 2.6 | Transient | Obligatory |  | Obligatory |
| 1e6e.A_B | [[14](#_ENREF_14)] | 2.3 | Obligatory | Obligatory |  | Obligatory |
| 1e6j.HL_P | [[14](#_ENREF_14)] | 3 | Transient | Transient |  | Transient |
| 1e8o.A_B | [[14](#_ENREF_14)] | 3.2 | Transient | Transient |  | Obligatory |
| 1e96.A_B | [[14](#_ENREF_14)] | 2.4 | Transient | Transient |  | Transient |
| 1e9z.A_B | [[14](#_ENREF_14)] | 3 | Obligatory | Obligatory |  | Obligatory |
| 1eai.A_C | [[14](#_ENREF_14)] | 2.4 | Transient | Transient |  | Transient |
| 1eay.A_C | [[14](#_ENREF_14)] | 2 | Transient | Transient |  | Transient |
| 1ebd.AB_C | [[14](#_ENREF_14)] | 2.6 | Transient | Transient |  | Transient |
| 1ebp.A_CD | [[14](#_ENREF_14)] | 2.8 | Transient | Transient |  | Transient |
| 1eer.A_B | [[14](#_ENREF_14)] | 1.9 | Transient | Transient |  | Transient |
| 1eex.A_B | [[14](#_ENREF_14)] | 1.7 | Obligatory | Obligatory |  | Obligatory |
| 1eex.A_G | [[14](#_ENREF_14)] | 1.7 | Obligatory | Obligatory |  | Obligatory |
| 1efu.A_B | [[14](#_ENREF_14)] | 2.5 | Transient | Transient |  | Obligatory |
| 1efv.A_B | [[31](#_ENREF_31)] | 2.1 | Obligatory | Obligatory |  | Obligatory |
| 1efx.ABC_D | [[14](#_ENREF_14)] | 3 | Transient | Transient |  | Transient |
| 1eg9.A_B | [[14](#_ENREF_14)] | 1.6 | Obligatory | Obligatory |  | Obligatory |
| 1eja.A_B | [[14](#_ENREF_14)] | 2.7 | Transient | Transient |  | Transient |
| 1ep3.A_B | [[14](#_ENREF_14)] | 2.1 | Obligatory | Transient | [[39](#_ENREF_39)] | Transient |
| 1es7.AC_B | [[14](#_ENREF_14)] | 2.9 | Transient | Transient |  | Obligatory |
| 1euv.A_B | [[14](#_ENREF_14)] | 1.6 | Transient | Transient |  | Transient |
| 1evt.A_C | [[14](#_ENREF_14)] | 2.8 | Transient | Transient |  | Transient |
| 1exb.A_E | [[14](#_ENREF_14)] | 2.1 | Obligatory | Transient | [[39](#_ENREF_39)] | Transient |
| 1ezv.C_F | [[14](#_ENREF_14)] | 2.3 | Obligatory | Obligatory |  | Obligatory |
| 1ezv.D_H | [[14](#_ENREF_14)] | 2.3 | Obligatory | Obligatory |  | Obligatory |
| 1ezv.E_XY | [[14](#_ENREF_14)] | 2.3 | Transient | Transient |  | Transient |
| 1ezx.AB_C | [[14](#_ENREF_14)] | 2.6 | Transient | Transient |  | Transient |
| 1f02.I_T | [[14](#_ENREF_14)] | 2.9 | Transient | Transient |  | Transient |
| 1f34.A_B | [[14](#_ENREF_14)] | 2.45 | Transient | Transient |  | Obligatory |
| 1f3u.A_B | [[14](#_ENREF_14)] | 1.7 | Obligatory | Obligatory |  | Obligatory |
| 1f3v.A_B | [[14](#_ENREF_14)] | 2 | Transient | Transient |  | Transient |
| 1f51.AB_E | [[14](#_ENREF_14)] | 3 | Transient | Transient |  | Obligatory |
| 1f5m.A_B | [[31](#_ENREF_31)] | 1.9 | Obligatory | Obligatory |  | Obligatory |
| 1f5q.A_B | [[31](#_ENREF_31)] | 2.5 | Transient | Transient |  | Transient |
| 1f60.A_B | [[14](#_ENREF_14)] | 1.67 | Transient | Transient |  | Obligatory |
| 1f6y.A_B | [[31](#_ENREF_31)] | 2.2 | Obligatory | Obligatory |  | Transient |
| 1f80.A_E | [[14](#_ENREF_14)] | 2.3 | Transient | Transient |  | Transient |
| 1f93.AB_EF | [[14](#_ENREF_14)] | 2.6 | Transient | Transient |  | Transient |
| 1fak.HL_T | [[14](#_ENREF_14)] | 2.1 | Transient | Transient |  | Transient |
| 1fbi.HL_X | [[14](#_ENREF_14)] | 3 | Transient | Transient |  | Transient |
| 1fbv.A_C | [[14](#_ENREF_14)] | 2.9 | Transient | Transient |  | Transient |
| 1fcd.A_C | [[14](#_ENREF_14)] | 2.53 | Obligatory | Obligatory |  | Obligatory |
| 1ffu.A_C | [[14](#_ENREF_14)] | 2.35 | Obligatory | Transient | [[71](#_ENREF_71)] | Transient |
| 1ffv.A_B | [[14](#_ENREF_14)] | 2.25 | Obligatory | Obligatory |  | Obligatory |
| 1fg9.AB_C | [[14](#_ENREF_14)] | 2.9 | Transient | Transient |  | Transient |
| 1fin.A_B | [[14](#_ENREF_14)] | 2.3 | Transient | Transient |  | Transient |
| 1fle.E_I | [[14](#_ENREF_14)] | 1.9 | Transient | Transient |  | Transient |
| 1fm0.D_E | [[14](#_ENREF_14)] | 1.45 | Obligatory | Transient | [[39](#_ENREF_39)] | Transient |
| 1fns.HL_A | [[14](#_ENREF_14)] | 2 | Transient | Transient |  | Transient |
| 1fq1.A_B | [[14](#_ENREF_14)] | 3 | Transient | Transient |  | Transient |
| 1fqj.A_C | [[14](#_ENREF_14)] | 2.02 | Transient | Transient |  | Transient |
| 1fqv.A_B | [[14](#_ENREF_14)] | 2.8 | Transient | Transient |  | Transient |
| 1fs0.E_G | [[14](#_ENREF_14)] | 2.1 | Obligatory | Obligatory |  | Obligatory |
| 1fsk.A_BC | [[14](#_ENREF_14)] | 2.9 | Transient | Transient |  | Transient |
| 1fxw.A_F | [[14](#_ENREF_14)] | 2.1 | Obligatory | Transient | [[39](#_ENREF_39)] | Transient |
| 1g0y.I_R | [[14](#_ENREF_14)] | 3 | Transient | Transient |  | Transient |
| 1g4y.R_B | [[14](#_ENREF_14)] | 1.6 | Transient | Transient |  | Transient |
| 1g73.AB_C | [[14](#_ENREF_14)] | 2 | Transient | Transient |  | Transient |
| 1g8k.A_B | [[14](#_ENREF_14)] | 1.64 | Obligatory | Obligatory |  | Obligatory |
| 1gaq.A_B | [[14](#_ENREF_14)] | 2.59 | Transient | Transient |  | Transient |
| 1gc1.C_G | [[14](#_ENREF_14)] | 2.5 | Transient | Transient |  | Transient |
| 1gcq.B_C | [[14](#_ENREF_14)] | 1.68 | Transient | Transient |  | Transient |
| 1gh6.A_B | [[14](#_ENREF_14)] | 3.2 | Transient | Transient |  | Transient |
| 1ghq.A_B | [[14](#_ENREF_14)] | 2.04 | Transient | Transient |  | Transient |
| 1gka.A_B | [[14](#_ENREF_14)] | 3.23 | Obligatory | Transient | [[39](#_ENREF_39)] | Transient |
| 1gl1.A_I | [[14](#_ENREF_14)] | 2.1 | Transient | Transient |  | Transient |
| 1gl4.A_B | [[14](#_ENREF_14)] | 2 | Transient | Transient |  | Transient |
| 1go3.E_F | [[14](#_ENREF_14)] | 1.75 | Obligatory | Obligatory |  | Obligatory |
| 1go4.A_G | [[14](#_ENREF_14)] | 2.05 | Transient | Transient |  | Obligatory |
| 1gp2.A_B | [[14](#_ENREF_14)] | 2.3 | Transient | Transient |  | Transient |
| 1gpe.A_B | [[31](#_ENREF_31)] | 1.8 | Obligatory | Obligatory |  | Transient |
| 1gpw.A_B | [[14](#_ENREF_14)] | 2.4 | Obligatory | Transient | [[72](#_ENREF_72)] | Transient |
| 1grn.A_B | [[14](#_ENREF_14)] | 2.1 | Transient | Transient |  | Transient |
| 1gvn.AC_B | [[14](#_ENREF_14)] | 1.95 | Transient | Transient |  | Transient |
| 1gxd.A_C | [[14](#_ENREF_14)] | 3.1 | Transient | Transient |  | Transient |
| 1gzs.A_B | [[14](#_ENREF_14)] | 2.3 | Transient | Transient |  | Obligatory |
| 1h2a.S_L | [[31](#_ENREF_31)] | 1.8 | Obligatory | Obligatory |  | Obligatory |
| 1h2k.A_S | [[14](#_ENREF_14)] | 2.15 | Transient | Transient |  | Transient |
| 1h2r.L_S | [[14](#_ENREF_14)] | 1.4 | Obligatory | Obligatory |  | Obligatory |
| 1h2v.C_Z | [[14](#_ENREF_14)] | 2 | Obligatory | Obligatory |  | Obligatory |
| 1h32.A_B | [[14](#_ENREF_14)] | 1.5 | Obligatory | Transient | [[73](#_ENREF_73)] | Transient |
| 1h4i.A_B | [[14](#_ENREF_14)] | 1.94 | Obligatory | Obligatory |  | Obligatory |
| 1h59.A_B | [[14](#_ENREF_14)] | 2.1 | Transient | Transient |  | Transient |
| 1h8e.A_D | [[14](#_ENREF_14)] | 2 | Obligatory | Obligatory |  | Obligatory |
| 1hcn.A_B | [[14](#_ENREF_14)] | 2.6 | Obligatory | Obligatory |  | Obligatory |
| 1he1.A_C | [[14](#_ENREF_14)] | 2 | Transient | Transient |  | Transient |
| 1hez.AB_E | [[14](#_ENREF_14)] | 2.7 | Transient | Transient |  | Transient |
| 1hfe.L_S | [[14](#_ENREF_14)] | 1.6 | Obligatory | Obligatory |  | Obligatory |
| 1hgx.A_B | [[31](#_ENREF_31)] | 1.9 | Obligatory | Transient | [[74](#_ENREF_74)] | Transient |
| 1hjr.A_B | [[31](#_ENREF_31)] | 2.5 | Obligatory | Transient | [[75](#_ENREF_75)] | Transient |
| 1hr6.AE_B | [[14](#_ENREF_14)] | 2.5 | Obligatory | Obligatory |  | Obligatory |
| 1hsa.A_B | [[14](#_ENREF_14)] | 2.1 | Obligatory | Transient | [[76](#_ENREF_76)] | Transient |
| 1hul.A_B | [[31](#_ENREF_31)] | 2.4 | Obligatory | Obligatory |  | Obligatory |
| 1hwg.A_BC | [[14](#_ENREF_14)] | 2.5 | Transient | Transient |  | Obligatory |
| 1hx1.A_B | [[14](#_ENREF_14)] | 1.9 | Transient | Transient |  | Transient |
| 1hzz.AB_C | [[14](#_ENREF_14)] | 2.5 | Obligatory | Obligatory |  | Obligatory |
| 1i1a.AB_CD | [[14](#_ENREF_14)] | 2.8 | Transient | Transient |  | Transient |
| 1i2m.A_B | [[14](#_ENREF_14)] | 1.76 | Transient | Transient |  | Obligatory |
| 1i3o.ABCD_E | [[14](#_ENREF_14)] | 2.7 | Transient | Transient |  | Obligatory |
| 1i4d.AB_D | [[14](#_ENREF_14)] | 2.5 | Transient | Transient |  | Transient |
| 1i4e.A_B | [[14](#_ENREF_14)] | 3 | Transient | Transient |  | Transient |
| 1i7w.A_B | [[14](#_ENREF_14)] | 2 | Transient | Transient |  | Transient |
| 1i85.B_D | [[14](#_ENREF_14)] | 3.2 | Transient | Transient |  | Transient |
| 1i8l.A_C | [[31](#_ENREF_31)] | 3 | Transient | Transient |  | Transient |
| 1i9r.ABC_HL | [[14](#_ENREF_14)] | 3.1 | Transient | Transient |  | Transient |
| 1ib1.AB_E | [[14](#_ENREF_14)] | 2.7 | Transient | Transient |  | Transient |
| 1ibr.A_B | [[14](#_ENREF_14)] | 2.3 | Transient | Transient |  | Obligatory |
| 1icf.AB_I | [[14](#_ENREF_14)] | 2 | Transient | Transient |  | Transient |
| 1ihf.A_B | [[14](#_ENREF_14)] | 2.2 | Obligatory | Obligatory |  | Obligatory |
| 1ijk.A_BC | [[14](#_ENREF_14)] | 2.6 | Transient | Transient |  | Transient |
| 1iod.AB_G | [[14](#_ENREF_14)] | 2.3 | Transient | Transient |  | Transient |
| 1iqd.AB_C | [[14](#_ENREF_14)] | 2 | Transient | Transient |  | Obligatory |
| 1ir1.A_S | [[14](#_ENREF_14)] | 1.8 | Obligatory | Obligatory |  | Obligatory |
| 1isa.A_B | [[31](#_ENREF_31)] | 1.8 | Obligatory | Obligatory |  | Transient |
| 1itb.A_B | [[14](#_ENREF_14)] | 2.5 | Transient | Transient |  | Obligatory |
| 1jb0.AB_C | [[14](#_ENREF_14)] | 2.5 | Obligatory | Obligatory |  | Obligatory |
| 1jb0.AB_D | [[14](#_ENREF_14)] | 2.5 | Obligatory | Obligatory |  | Obligatory |
| 1jb0.AB_E | [[14](#_ENREF_14)] | 2.5 | Obligatory | Obligatory |  | Obligatory |
| 1jb0.C_D | [[14](#_ENREF_14)] | 2.5 | Obligatory | Obligatory |  | Obligatory |
| 1jb0.C_E | [[14](#_ENREF_14)] | 2.5 | Obligatory | Transient | [[39](#_ENREF_39)] | Transient |
| 1jb7.A_B | [[14](#_ENREF_14)] | 1.86 | Obligatory | Obligatory |  | Obligatory |
| 1jch.A_B | [[14](#_ENREF_14)] | 3.02 | Transient | Transient |  | Transient |
| 1jiw.P_I | [[14](#_ENREF_14)] | 1.74 | Transient | Transient |  | Obligatory |
| 1jk0.A_B | [[14](#_ENREF_14)] | 2.8 | Obligatory | Transient | [[77](#_ENREF_77)] | Transient |
| 1jk8.A_B | [[14](#_ENREF_14)] | 2.4 | Obligatory | Obligatory |  | Obligatory |
| 1jkj.A_B | [[14](#_ENREF_14)] | 2.35 | Obligatory | Obligatory |  | Obligatory |
| 1jkm.A_B | [[31](#_ENREF_31)] | 1.85 | Obligatory | Obligatory |  | Obligatory |
| 1jma.A_B | [[14](#_ENREF_14)] | 2.65 | Transient | Transient |  | Transient |
| 1jmx.A_G | [[14](#_ENREF_14)] | 1.9 | Obligatory | Obligatory |  | Obligatory |
| 1jmz.AG_B | [[14](#_ENREF_14)] | 2 | Obligatory | Obligatory |  | Obligatory |
| 1jnr.A_B | [[14](#_ENREF_14)] | 1.6 | Obligatory | Obligatory |  | Obligatory |
| 1jro.A_BD | [[14](#_ENREF_14)] | 2.7 | Obligatory | Obligatory |  | Obligatory |
| 1jsu.AB_C | [[14](#_ENREF_14)] | 2.3 | Transient | Transient |  | Obligatory |
| 1jtg.A_B | [[14](#_ENREF_14)] | 1.73 | Transient | Transient |  | Transient |
| 1jv2.A_B | [[14](#_ENREF_14)] | 3.1 | Obligatory | Obligatory |  | Obligatory |
| 1jw9.B_D | [[14](#_ENREF_14)] | 1.7 | Transient | Transient |  | Obligatory |
| 1jwh.A_CD | [[14](#_ENREF_14)] | 3.1 | Obligatory | Transient | [[78](#_ENREF_78)] | Transient |
| 1k3u.A_B | [[14](#_ENREF_14)] | 1.7 | Obligatory | Obligatory |  | Obligatory |
| 1k3z.AB_D | [[14](#_ENREF_14)] | 2.5 | Transient | Transient |  | Obligatory |
| 1k4c.AB_C | [[14](#_ENREF_14)] | 2 | Transient | Transient |  | Obligatory |
| 1k5d.A_B | [[14](#_ENREF_14)] | 2.7 | Transient | Transient |  | Obligatory |
| 1k5d.A_C | [[14](#_ENREF_14)] | 2.7 | Transient | Transient |  | Obligatory |
| 1k8k.A_B | [[14](#_ENREF_14)] | 2 | Obligatory | Transient | [[79](#_ENREF_79)] | Transient |
| 1k8k.A_E | [[14](#_ENREF_14)] | 2 | Obligatory | Transient | [[79](#_ENREF_79)] | Transient |
| 1k8k.B_F | [[14](#_ENREF_14)] | 2 | Obligatory | Transient | [[39](#_ENREF_39)] | Transient |
| 1k8k.D_F | [[14](#_ENREF_14)] | 2 | Obligatory | Transient | [[79](#_ENREF_79)] | Transient |
| 1k90.A_D | [[14](#_ENREF_14)] | 2.75 | Transient | Transient |  | Obligatory |
| 1kac.A_B | [[14](#_ENREF_14)] | 2.6 | Transient | Transient |  | Transient |
| 1kcg.AB_C | [[14](#_ENREF_14)] | 2.6 | Transient | Transient |  | Transient |
| 1kfu.L_S | [[14](#_ENREF_14)] | 2.5 | Obligatory | Obligatory |  | Obligatory |
| 1kgy.A_E | [[14](#_ENREF_14)] | 2.7 | Transient | Transient |  | Transient |
| 1ki1.A_B | [[14](#_ENREF_14)] | 2.3 | Transient | Transient |  | Transient |
| 1kkl.ABC_H | [[14](#_ENREF_14)] | 2.8 | Transient | Transient |  | Obligatory |
| 1kmi.Y_Z | [[14](#_ENREF_14)] | 2.9 | Transient | Transient |  | Transient |
| 1kpe.A_B | [[31](#_ENREF_31)] | 1.8 | Obligatory | Obligatory |  | Obligatory |
| 1kqf.A_B | [[14](#_ENREF_14)] | 1.6 | Obligatory | Obligatory |  | Obligatory |
| 1kqf.B_C | [[14](#_ENREF_14)] | 1.6 | Obligatory | Obligatory |  | Transient |
| 1ktd.A_B | [[14](#_ENREF_14)] | 2.4 | Obligatory | Obligatory |  | Obligatory |
| 1kxp.A_D | [[14](#_ENREF_14)] | 2.1 | Transient | Transient |  | Transient |
| 1kxt.A_B | [[14](#_ENREF_14)] | 2 | Transient | Transient |  | Obligatory |
| 1kyo.O_W | [[14](#_ENREF_14)] | 2.97 | Transient | Transient |  | Transient |
| 1kzy.A_C | [[14](#_ENREF_14)] | 2.5 | Transient | Transient |  | Transient |
| 1l0o.AB_C | [[14](#_ENREF_14)] | 2.9 | Transient | Transient |  | Transient |
| 1l6x.A_B | [[14](#_ENREF_14)] | 1.65 | Transient | Transient |  | Transient |
| 1l7v.AB_C | [[14](#_ENREF_14)] | 3.2 | Obligatory | Transient | [[39](#_ENREF_39)] | Transient |
| 1l9j.C_HLM | [[14](#_ENREF_14)] | 3.25 | Obligatory | Obligatory |  | Obligatory |
| 1lb1.A_B | [[14](#_ENREF_14)] | 2.81 | Transient | Transient |  | Transient |
| 1ld8.A_B | [[14](#_ENREF_14)] | 1.8 | Obligatory | Obligatory |  | Obligatory |
| 1ldj.A_B | [[14](#_ENREF_14)] | 3 | Obligatory | Obligatory |  | Obligatory |
| 1li1.AB_C | [[14](#_ENREF_14)] | 1.9 | Obligatory | Obligatory |  | Obligatory |
| 1lk3.A_HL | [[14](#_ENREF_14)] | 1.91 | Transient | Transient |  | Transient |
| 1lpb.A_B | [[14](#_ENREF_14)] | 2.46 | Transient | Transient |  | Obligatory |
| 1lti.AC_DEHFG | [[14](#_ENREF_14)] | 2.13 | Obligatory | Transient | [[80](#_ENREF_80)] | Transient |
| 1luc.A_B | [[31](#_ENREF_31)] | 1.5 | Obligatory | Obligatory |  | Obligatory |
| 1m10.A_B | [[14](#_ENREF_14)] | 3.1 | Transient | Transient |  | Transient |
| 1m1e.A_B | [[14](#_ENREF_14)] | 2.1 | Transient | Transient |  | Transient |
| 1m4u.A_L | [[14](#_ENREF_14)] | 2.42 | Transient | Transient |  | Transient |
| 1mah.A_F | [[14](#_ENREF_14)] | 3.2 | Transient | Transient |  | Transient |
| 1mbu.A_C | [[14](#_ENREF_14)] | 2.3 | Transient | Transient |  | Transient |
| 1mct.A_I | [[31](#_ENREF_31)] | 1.6 | Transient | Transient |  | Transient |
| 1mjg.AB_M | [[14](#_ENREF_14)] | 2.2 | Obligatory | Obligatory |  | Obligatory |
| 1mka.A_B | [[31](#_ENREF_31)] | 2 | Obligatory | Obligatory |  | Obligatory |
| 1ml0.A_D | [[14](#_ENREF_14)] | 2.8 | Transient | Transient |  | Transient |
| 1mr1.A_D | [[14](#_ENREF_14)] | 2.85 | Transient | Transient |  | Transient |
| 1mro.A_B | [[14](#_ENREF_14)] | 1.16 | Obligatory | Transient | [[20](#_ENREF_20)] | Transient |
| 1mro.A_C | [[14](#_ENREF_14)] | 1.16 | Obligatory | Obligatory |  | Obligatory |
| 1mro.B_C | [[14](#_ENREF_14)] | 1.16 | Obligatory | Obligatory |  | Obligatory |
| 1msp.A_B | [[31](#_ENREF_31)] | 2.5 | Obligatory | Obligatory |  | Transient |
| 1n2c.AB_EF | [[14](#_ENREF_14)] | 3 | Transient | Transient |  | Obligatory |
| 1nbf.A_D | [[14](#_ENREF_14)] | 2.3 | Transient | Transient |  | Obligatory |
| 1nbw.AC_B | [[14](#_ENREF_14)] | 2.4 | Obligatory | Obligatory |  | Obligatory |
| 1nf5.A_B | [[14](#_ENREF_14)] | 2 | Transient | Transient |  | Transient |
| 1noc.A_B | [[14](#_ENREF_14)] | 2.6 | Transient | Transient |  | Transient |
| 1nse.A_B | [[31](#_ENREF_31)] | 1.9 | Obligatory | Obligatory |  | Obligatory |
| 1nsn.HL_S | [[14](#_ENREF_14)] | 2.8 | Transient | Transient |  | Transient |
| 1nsy.A_B | [[31](#_ENREF_31)] | 2 | Obligatory | Obligatory |  | Obligatory |
| 1o6s.A_B | [[14](#_ENREF_14)] | 1.8 | Transient | Transient |  | Transient |
| 1one.A_B | [[31](#_ENREF_31)] | 1.8 | Obligatory | Obligatory |  | Obligatory |
| 1osp.HL_O | [[14](#_ENREF_14)] | 1.95 | Transient | Transient |  | Transient |
| 1pdk.A_B | [[31](#_ENREF_31)] | 2.4 | Transient | Transient |  | Obligatory |
| 1pnk.A_B | [[31](#_ENREF_31)] | 1.9 | Obligatory | Obligatory |  | Obligatory |
| 1poi.A_B | [[14](#_ENREF_14)] | 2.5 | Obligatory | Obligatory |  | Obligatory |
| 1prc.C_HLM | [[14](#_ENREF_14)] | 2.3 | Obligatory | Obligatory |  | Obligatory |
| 1pvu.A_B | [[31](#_ENREF_31)] | 2.4 | Obligatory | Transient | [[81](#_ENREF_81)] | Transient |
| 1qae.A_B | [[31](#_ENREF_31)] | 2.05 | Obligatory | Obligatory |  | Transient |
| 1qav.A_B | [[14](#_ENREF_14)] | 1.9 | Transient | Transient |  | Transient |
| 1qax.A_B | [[31](#_ENREF_31)] | 2.8 | Obligatory | Obligatory |  | Obligatory |
| 1qbi.A_B | [[31](#_ENREF_31)] | 1.72 | Obligatory | Obligatory |  | Transient |
| 1qdl.A_B | [[14](#_ENREF_14)] | 2.5 | Obligatory | Obligatory |  | Obligatory |
| 1qfe.A_B | [[31](#_ENREF_31)] | 2.1 | Obligatory | Obligatory |  | Transient |
| 1qfh.A_B | [[31](#_ENREF_31)] | 2.2 | Obligatory | Obligatory |  | Obligatory |
| 1qfu.AB_HL | [[14](#_ENREF_14)] | 2.8 | Transient | Transient |  | Transient |
| 1qfw.AB_IM | [[14](#_ENREF_14)] | 3.5 | Transient | Transient |  | Transient |
| 1qgw.A_C | [[14](#_ENREF_14)] | 1.63 | Obligatory | Obligatory |  | Obligatory |
| 1qi9.A_B | [[31](#_ENREF_31)] | 2.05 | Obligatory | Obligatory |  | Obligatory |
| 1qkz.A_HL | [[14](#_ENREF_14)] | 1.95 | Transient | Transient |  | Transient |
| 1qlb.B_C | [[14](#_ENREF_14)] | 2.33 | Obligatory | Obligatory |  | Obligatory |
| 1qo0.A_DE | [[14](#_ENREF_14)] | 2.25 | Transient | Transient |  | Transient |
| 1qor.A_B | [[31](#_ENREF_31)] | 2.2 | Obligatory | Obligatory |  | Transient |
| 1qqj.A_B | [[31](#_ENREF_31)] | 1.55 | Obligatory | Obligatory |  | Obligatory |
| 1qu7.A_B | [[31](#_ENREF_31)] | 2.6 | Obligatory | Obligatory |  | Obligatory |
| 1raf.A_BD | [[14](#_ENREF_14)] | 2.5 | Obligatory | Transient | [[39](#_ENREF_39)] | Transient |
| 1req.A_B | [[14](#_ENREF_14)] | 2 | Obligatory | Obligatory |  | Obligatory |
| 1rlb.ABCD_E | [[14](#_ENREF_14)] | 3.1 | Transient | Transient |  | Transient |
| 1sbb.A_B | [[14](#_ENREF_14)] | 2.4 | Transient | Transient |  | Transient |
| 1sgf.A_BY | [[14](#_ENREF_14)] | 3.15 | Obligatory | Transient | [[20](#_ENREF_20)] | Transient |
| 1smf.E_I | [[14](#_ENREF_14)] | 2.1 | Transient | Transient |  | Transient |
| 1smt.A_B | [[31](#_ENREF_31)] | 2.2 | Obligatory | Obligatory |  | Obligatory |
| 1sox.A_B | [[31](#_ENREF_31)] | 1.9 | Obligatory | Obligatory |  | Obligatory |
| 1spp.A_B | [[14](#_ENREF_14)] | 2.4 | Obligatory | Transient | [[82](#_ENREF_82)] | Transient |
| 1spu.A_B | [[31](#_ENREF_31)] | 2 | Obligatory | Obligatory |  | Obligatory |
| 1stf.E_I | [[14](#_ENREF_14)] | 2.37 | Transient | Transient |  | Transient |
| 1t7p.A_B | [[14](#_ENREF_14)] | 2.2 | Transient | Transient |  | Transient |
| 1tab.I_E | [[31](#_ENREF_31)] | 2.3 | Transient | Transient |  | Transient |
| 1tbg.A_E | [[14](#_ENREF_14)] | 2.1 | Obligatory | Obligatory |  | Obligatory |
| 1tgs.Z_I | [[31](#_ENREF_31)] | 1.8 | Transient | Transient |  | Transient |
| 1tmq.A_B | [[14](#_ENREF_14)] | 2.5 | Transient | Transient |  | Obligatory |
| 1toc.R_AB | [[14](#_ENREF_14)] | 3.1 | Transient | Transient |  | Obligatory |
| 1trk.A_B | [[31](#_ENREF_31)] | 2 | Obligatory | Obligatory |  | Obligatory |
| 1tx4.A_B | [[31](#_ENREF_31)] | 1.65 | Transient | Transient |  | Transient |
| 1udi.I_E | [[31](#_ENREF_31)] | 2.7 | Transient | Transient |  | Transient |
| 1ugh.I_E | [[14](#_ENREF_14)] | 1.9 | Transient | Transient |  | Transient |
| 1vcb.A_B | [[14](#_ENREF_14)] | 2.7 | Obligatory | Obligatory |  | Obligatory |
| 1vfr.A_B | [[31](#_ENREF_31)] | 1.8 | Obligatory | Obligatory |  | Obligatory |
| 1vhi.A_B | [[31](#_ENREF_31)] | 2.5 | Obligatory | Obligatory |  | Obligatory |
| 1viw.A_B | [[31](#_ENREF_31)] | 3 | Transient | Transient |  | Obligatory |
| 1vkx.A_B | [[14](#_ENREF_14)] | 2.9 | Obligatory | Transient | [[83](#_ENREF_83)] | Transient |
| 1vlt.A_B | [[31](#_ENREF_31)] | 2.2 | Obligatory | Transient | [[84](#_ENREF_84)] | Transient |
| 1vok.A_B | [[31](#_ENREF_31)] | 2.1 | Obligatory | Transient | [[85](#_ENREF_85)] | Transient |
| 1vsg.A_B | [[31](#_ENREF_31)] | 2.9 | Obligatory | Obligatory |  | Obligatory |
| 1wej.HL_F | [[14](#_ENREF_14)] | 1.8 | Transient | Transient |  | Transient |
| 1wgj.A_B | [[31](#_ENREF_31)] | 2 | Obligatory | Transient | [[86](#_ENREF_86)] | Transient |
| 1wq1.G_R | [[14](#_ENREF_14)] | 2.5 | Transient | Transient |  | Transient |
| 1xdt.R_T | [[14](#_ENREF_14)] | 2.65 | Transient | Transient |  | Transient |
| 1xik.A_B | [[31](#_ENREF_31)] | 1.7 | Obligatory | Obligatory |  | Obligatory |
| 1xso.A_B | [[31](#_ENREF_31)] | 1.49 | Obligatory | Obligatory |  | Obligatory |
| 1ycs.A_B | [[14](#_ENREF_14)] | 2.2 | Transient | Transient |  | Transient |
| 1ypi.A_B | [[31](#_ENREF_31)] | 1.9 | Obligatory | Obligatory |  | Transient |
| 1ytf.BC_D | [[14](#_ENREF_14)] | 2.5 | Obligatory | Obligatory |  | Obligatory |
| 1zbd.A_B | [[14](#_ENREF_14)] | 2.6 | Transient | Transient |  | Transient |
| 2aai.A_B | [[31](#_ENREF_31)] | 2.5 | Obligatory | Transient | [[39](#_ENREF_39)] | Transient |
| 2ae2.A_B | [[31](#_ENREF_31)] | 1.9 | Obligatory | Obligatory |  | Transient |
| 2arc.A_B | [[31](#_ENREF_31)] | 1.5 | Obligatory | Obligatory |  | Transient |
| 2btc.I_E | [[14](#_ENREF_14)] | 1.5 | Transient | Transient |  | Transient |
| 2btf.P_A | [[14](#_ENREF_14)] | 2.55 | Transient | Transient |  | Obligatory |
| 2gsa.A_B | [[31](#_ENREF_31)] | 2.4 | Obligatory | Obligatory |  | Obligatory |
| 2hdh.A_B | [[31](#_ENREF_31)] | 2.2 | Obligatory | Obligatory |  | Transient |
| 2hhm.A_B | [[31](#_ENREF_31)] | 2.1 | Obligatory | Obligatory |  | Obligatory |
| 2hmi.AB_CD | [[14](#_ENREF_14)] | 2.8 | Transient | Transient |  | Transient |
| 2jel.HL_P | [[14](#_ENREF_14)] | 2.5 | Transient | Transient |  | Transient |
| 2min.A_B | [[14](#_ENREF_14)] | 2.03 | Obligatory | Obligatory |  | Obligatory |
| 2mta.A_C | [[14](#_ENREF_14)] | 2.4 | Transient | Transient |  | Transient |
| 2mta.A_HL | [[14](#_ENREF_14)] | 2.4 | Transient | Transient |  | Obligatory |
| 2mta.H_L | [[14](#_ENREF_14)] | 2.4 | Obligatory | Obligatory |  | Obligatory |
| 2nac.A_B | [[31](#_ENREF_31)] | 1.8 | Obligatory | Obligatory |  | Obligatory |
| 2pcc.A_B | [[14](#_ENREF_14)] | 2.3 | Transient | Transient |  | Transient |
| 2pfl.A_B | [[31](#_ENREF_31)] | 2.9 | Obligatory | Obligatory |  | Transient |
| 2ptc.I_E | [[31](#_ENREF_31)] | 1.9 | Transient | Transient |  | Transient |
| 2sic.E_I | [[14](#_ENREF_14)] | 1.8 | Transient | Transient |  | Transient |
| 2utg.A_B | [[31](#_ENREF_31)] | 1.64 | Obligatory | Obligatory |  | Obligatory |
| 3gtu.A_B | [[14](#_ENREF_14)] | 2.8 | Obligatory | Obligatory |  | Obligatory |
| 3pce.A_M | [[14](#_ENREF_14)] | 2.06 | Obligatory | Obligatory |  | Obligatory |
| 3ygs.P_C | [[14](#_ENREF_14)] | 2.5 | Transient | Transient |  | Transient |
| 4htc.HL_I | [[14](#_ENREF_14)] | 2.3 | Transient | Transient |  | Transient |
| 4mdh.A_B | [[31](#_ENREF_31)] | 2.5 | Obligatory | Obligatory |  | Obligatory |
| 4rub.AD_T | [[14](#_ENREF_14)] | 2.7 | Obligatory | Obligatory |  | Obligatory |
| 4sgb.I_E | [[14](#_ENREF_14)] | 2.1 | Transient | Transient |  | Transient |
| 7cei.A_B | [[14](#_ENREF_14)] | 2.3 | Transient | Transient |  | Transient |

The original references as well as the references used to update the interaction type are given. DynaFace predictions are also included.
