# Supplementary material for: DynaFace: Discrimination between Obligatory and Non-obligatory Protein-Protein Interactions Based on the Complex’s Dynamics
Source: PLoS Comput Biol. 2015 Oct 27;11(10):e1004461. doi: 10.1371/journal.pcbi.1004461 (PMC4623975; doi:10.1371/journal.pcbi.1004461)
Supplement: S2 Table — “Model ID”–the PrePPI index number of the predicted dimer model. “Probability”- the PrePPI prediction likelihood: high, low, very low. “Template ID”–the PDB accession number of the template PrePPI used to model the dimer. “Template Chain IDs”–the chains PrePPI used to model the structure. “Model”–DynaFace prediction of the interface type of the model structure: obligatory vs. non-obligatory. “Template”–DynaFace prediction of the interface of the template. (DOCX) [file pcbi.1004461.s006.docx]

**S2 Table. Dataset of 85 template structures and the PrePPI structural models predicted based on these template structures [**[**6**](#_ENREF_6)**] along with their DynaFace predictions.**

| **Model ID** | **Probability** | **Template ID** | **Template Chain IDs** | | **PITPRED** | | |
| --- | --- | --- | --- | --- | --- | --- | --- |
|  |  |  |  |  | **Model** | | **Template** |
| P00352_O75891 | high | 2O2P | A | B | Obligatory | Obligatory | |
| P00403_P00395 | high | 1V55 | A | B | Obligatory | Obligatory | |
| P01137_O60383 | high | 1KLD | A | B | Obligatory | Obligatory | |
| P01137_O95393 | high | 1KLD | A | B | Obligatory | Obligatory | |
| P01137_O95972 | high | 1KLD | A | B | Obligatory | Obligatory | |
| P01215_A6NKQ9 | high | 1QFW | A | B | Obligatory | Obligatory | |
| P06702_P04271 | high | 1PSB | A | B | Obligatory | Obligatory | |
| P07864_P00338 | high | 9LDT | A | B | Obligatory | Obligatory | |
| P09493_P06753 | high | 1C1G | A | B | Obligatory | Obligatory | |
| P09529_P01137 | high | 1KLD | A | B | Obligatory | Obligatory | |
| P17174_P00505 | high | 1AJR | A | B | Transient | Obligatory | |
| P18075_P01137 | high | 1KLD | A | B | Transient | Obligatory | |
| P25815_P04271 | high | 1OZO | A | B | Obligatory | Obligatory | |
| P25815_P05109 | high | 1OZO | A | B | Obligatory | Obligatory | |
| P26447_P04271 | high | 1PSB | A | B | Obligatory | Obligatory | |
| P29508_P01009 | high | 8API | A | B | Obligatory | Transient | |
| P30038_P00352 | high | 1BXS | A | B | Obligatory | Obligatory | |
| P30038_P05091 | high | 1O04 | A | B | Obligatory | Obligatory | |
| P30837_O75891 | high | 2O2P | A | B | Obligatory | Obligatory | |
| P30837_P00352 | high | 1BXS | A | B | Obligatory | Obligatory | |
| P30837_P05091 | high | 1O04 | A | B | Obligatory | Obligatory | |
| P35237_P01009 | high | 8API | A | B | Obligatory | Transient | |
| P43026_P01137 | high | 1KLD | A | B | Obligatory | Obligatory | |
| P47895_O75891 | high | 2O2P | A | B | Obligatory | Obligatory | |
| P48594_P01009 | high | 8API | A | B | Obligatory | Transient | |
| P49189_O75891 | high | 2O2P | A | B | Obligatory | Obligatory | |
| P49189_P00352 | high | 1BXS | A | B | Obligatory | Obligatory | |
| P49189_P05091 | high | 1O04 | A | B | Obligatory | Obligatory | |
| P49419_O75891 | high | 2O2P | A | B | Obligatory | Obligatory | |
| P49419_O94788 | high | 2J6L | E | F | Obligatory | Obligatory | |
| P49419_P00352 | high | 2J6L | A | B | Obligatory | Obligatory | |
| P49419_P05091 | high | 1O04 | A | B | Obligatory | Obligatory | |
| P49419_P30837 | high | 2J6L | A | B | Obligatory | Obligatory | |
| P49419_P47895 | high | 2J6L | A | B | Obligatory | Obligatory | |
| P49419_P49189 | high | 2J6L | A | B | Obligatory | Obligatory | |
| P50453_P01009 | high | 8API | A | B | Obligatory | Transient | |
| P51649_O75891 | high | 2O2P | A | B | Obligatory | Obligatory | |
| P51649_P00352 | high | 1BXS | A | B | Obligatory | Obligatory | |
| P51649_P05091 | high | 1O04 | A | B | Obligatory | Obligatory | |
| P51649_P49419 | high | 2J6L | A | B | Obligatory | Obligatory | |
| P52907_P47756 | high | 1IZN | A | B | Obligatory | Obligatory | |
| P53609_P49354 | high | 1S64 | A | B | Obligatory | Obligatory | |
| P55084_P09110 | high | 2IIK | A | B | Obligatory | Obligatory | |
| P55084_P24752 | high | 2IB8 | A | B | Obligatory | Obligatory | |
| P55107_P01137 | high | 1KLD | A | B | Transient | Obligatory | |
| P58166_P01137 | high | 1KLD | A | B | Obligatory | Obligatory | |
| P60903_P25815 | high | 1OZO | A | B | Obligatory | Obligatory | |
| Q02252_O75891 | high | 2O2P | A | B | Obligatory | Obligatory | |
| Q02252_P00352 | high | 1BXS | A | B | Obligatory | Obligatory | |
| Q02252_P05091 | high | 1O04 | A | B | Obligatory | Obligatory | |
| Q02252_P49419 | high | 2J6L | A | B | Obligatory | Obligatory | |
| Q16881_P00390 | high | 2GH5 | A | B | Obligatory | Obligatory | |
| Q3SY69_P49419 | high | 2J6L | A | B | Obligatory | Obligatory | |
| Q6KF10_P01137 | high | 1KLD | A | B | Obligatory | Obligatory | |
| Q6NT52_P01215 | high | 1QFW | A | B | Obligatory | Obligatory | |
| Q6NXT2_P62805 | high | 1P3K | A | B | Obligatory | Obligatory | |
| Q6YP21_Q16773 | high | 3FVX | A | B | Obligatory | Obligatory | |
| Q6ZMR3_P00338 | high | 1I10 | A | B | Obligatory | Obligatory | |
| Q71DI3_P62805 | high | 1P3K | A | B | Obligatory | Obligatory | |
| Q7Z4P5_P01137 | high | 1KLD | A | B | Obligatory | Obligatory | |
| Q86WD7_P01009 | high | 8API | A | B | Obligatory | Transient | |
| Q92781_P14061 | high | 1FDU | A | B | Obligatory | Obligatory | |
| Q96KX2_P47756 | high | 1IZN | A | B | Transient | Obligatory | |
| Q99584_P04271 | high | 1PSB | A | B | Obligatory | Obligatory | |
| Q99584_P25815 | high | 1OZO | A | B | Obligatory | Obligatory | |
| Q99962_Q17R89 | high | 1ZWW | A | B | Obligatory | Obligatory | |
| Q9BWD1_P24752 | high | 2IB8 | C | D | Obligatory | Obligatory | |
| Q9BY49_Q16698 | high | 1W8D | A | B | Obligatory | Obligatory | |
| Q9BYV1_P04181 | high | 2OAT | A | B | Obligatory | Obligatory | |
| Q9GZM7_P07858 | high | 1CSB | D | E | Obligatory | Transient | |
| Q9H2A2_O75891 | high | 2O2P | A | B | Obligatory | Obligatory | |
| Q9H2A2_P00352 | high | 1BXS | A | B | Transient | Obligatory | |
| Q9H2A2_P05091 | high | 1O04 | A | B | Obligatory | Obligatory | |
| Q9H2A2_P49419 | high | 2J6L | A | B | Transient | Obligatory | |
| Q9HCY8_P04271 | high | 1PSB | A | B | Obligatory | Obligatory | |
| Q9HCY8_P25815 | high | 1OZO | A | B | Obligatory | Obligatory | |
| Q9NNW7_P00390 | high | 2GH5 | A | B | Obligatory | Obligatory | |
| Q9NR33_O60814 | high | 1EQZ | A | B | Obligatory | Obligatory | |
| Q9NR33_P06899 | high | 1EQZ | A | B | Obligatory | Obligatory | |
| Q9NR33_P23527 | high | 1EQZ | A | B | Obligatory | Obligatory | |
| Q9NR33_P33778 | high | 1EQZ | A | B | Obligatory | Obligatory | |
| Q9NR33_P57053 | high | 1EQZ | A | B | Obligatory | Obligatory | |
| Q9NR33_P58876 | high | 1EQZ | A | B | Obligatory | Obligatory | |
| Q9NR33_P62807 | high | 1EQZ | A | B | Obligatory | Obligatory | |
| Q9NR33_Q16778 | high | 1EQZ | A | B | Obligatory | Obligatory | |
| Q9NR33_Q5QNW6 | high | 1EQZ | A | B | Obligatory | Obligatory | |
| Q9NR33_Q8N257 | high | 1EQZ | A | B | Obligatory | Obligatory | |
| Q9NR33_Q93079 | high | 1EQZ | A | B | Obligatory | Obligatory | |
| Q9NR33_Q96A08 | high | 1EQZ | A | B | Obligatory | Obligatory | |
| Q9NR33_Q99877 | high | 1EQZ | A | B | Obligatory | Obligatory | |
| Q9NR33_Q99879 | high | 1EQZ | A | B | Obligatory | Obligatory | |
| Q9NR33_Q99880 | high | 1EQZ | A | B | Obligatory | Obligatory | |
| Q9NRD8_P05164 | high | 1DNW | A | C | Obligatory | Obligatory | |
| Q9NRD9_P05164 | high | 1DNW | A | C | Obligatory | Obligatory | |
| Q9NYR8_P14061 | high | 1FDU | A | B | Obligatory | Obligatory | |
| Q9UBQ7_P56545 | high | 2Q50 | A | B | Obligatory | Obligatory | |
| Q9UBQ7_Q13363 | high | 2Q50 | A | B | Obligatory | Obligatory | |
| Q9UK55_P01009 | high | 8API | A | B | Obligatory | Transient | |
| Q9Y4D1_Q86T65 | high | 2Z6E | A | B | Transient | Obligatory | |
| Q9Y4D1_Q96PY5 | high | 2Z6E | A | B | Obligatory | Obligatory | |
| P63092_P50148 | low | 1AZT | A | B | Transient | Transient | |
| P02671_O15123 | low | 1FZD | A | E | Obligatory | DID NOT RUN | |
| P02671_O43827 | low | 1FZD | A | E | Obligatory | DID NOT RUN | |
| P22105_P02671 | low | 1FZD | A | E | Obligatory | DID NOT RUN | |
| P24821_P02671 | low | 1FZD | A | E | Obligatory | DID NOT RUN | |
| P43250_O96017 | low | 2ACX | A | B | DID NOT RUN | DID NOT RUN | |
| P01861_P01842 | low | 1MCQ | A | B | Obligatory | Obligatory | |
| Q00872_P01764 | low | 1OL0 | A | B | Transient | Obligatory | |
| P49792_P11233 | low | 1RRP | A | B | Transient | Obligatory | |
| P13489_O60810 | low | 1Z7X | W | Y | Transient | Obligatory | |
| P05230_O43581 | low | 2K4A | A | B | Obligatory | Transient | |
| P49798_O14775 | low | 2PBI | A | B | Transient | Obligatory | |
| Q04759_P10644 | low | 2QCS | A | B | Transient | Obligatory | |
| P61160_A5A3E0 | low | 3B63 | K | L | Transient | Transient | |
| P47736_P08134 | low | 3BRW | B | D | Obligatory | Transient | |
| P47736_P15153 | low | 3BRW | B | D | Obligatory | Transient | |
| P47736_P20337 | low | 3BRW | B | D | Obligatory | Transient | |
| P51153_P47736 | low | 3BRW | B | D | Obligatory | Transient | |
| P60763_P47736 | low | 3BRW | B | D | Obligatory | Transient | |
| P61026_P47736 | low | 3BRW | B | D | Obligatory | Transient | |
| P61586_P47736 | low | 3BRW | B | D | Obligatory | Transient | |
| P63000_P47736 | low | 3BRW | B | D | Obligatory | Transient | |
| P22891_P05546 | low | 3F1S | A | B | Obligatory | Transient | |
| P22891_P08697 | low | 3F1S | A | B | Obligatory | Transient | |
| P16144_A2RUH7 | low | 3F7Q | A | B | Transient | Transient | |
| P16144_O15394 | low | 3F7Q | A | B | Obligatory | Transient | |
| P16144_O60500 | low | 3F7Q | A | B | Obligatory | Transient | |
| P22105_P16144 | low | 3F7Q | A | B | Obligatory | Transient | |
| P30281_P20248 | low | 3G33 | B | D | Obligatory | Transient | |
| P41002_P30281 | low | 3G33 | B | D | Obligatory | Transient | |
| P78396_P30281 | low | 3G33 | B | D | Obligatory | Transient | |
| P63092_O95837 | low | 1AZT | A | B | Transient | Transient | |
| P63092_P09471 | low | 1AZT | A | B | Transient | Transient | |
| P63092_P11488 | low | 1AZT | A | B | Transient | Transient | |
| P63092_P29992 | low | 1AZT | A | B | Transient | Transient | |
| Q03113_P63092 | low | 1AZT | A | B | Transient | Transient | |
| P62820_O15085 | low | 1XCG | A | B | Transient | Transient | |
| P78386_P51692 | low | 1Y1U | A | B | Transient | Transient | |
| P55854_P35241 | low | 2D10 | A | D | Transient | Transient | |
| P61956_P35241 | low | 2D10 | A | D | Transient | Transient | |
| P62988_P35241 | low | 2D10 | A | D | Transient | Transient | |
| P60709_O95551 | low | 2D1K | A | B | Transient | Transient | |
| P62736_O95551 | low | 2D1K | A | B | Transient | Transient | |
| P63261_O95551 | low | 2D1K | A | B | Transient | Transient | |
| P63267_O95551 | low | 2D1K | A | B | Transient | Transient | |
| P68032_O95551 | low | 2D1K | A | B | Transient | Transient | |
| P68133_O95551 | low | 2D1K | A | B | Transient | Transient | |
| P05230_O14795 | low | 2K4A | A | B | Transient | Transient | |
| P21579_O43320 | low | 2K4A | A | B | Transient | Transient | |
| P21579_O76093 | low | 2K4A | A | B | Transient | Transient | |
| P21579_O95750 | low | 2K4A | A | B | Transient | Transient | |
| P21579_P08620 | low | 2K4A | A | B | Transient | Transient | |
| P21579_P11487 | low | 2K4A | A | B | Transient | Transient | |
| P21579_P12034 | low | 2K4A | A | B | Transient | Transient | |
| P31371_P21579 | low | 2K4A | A | B | Transient | Transient | |
| P55075_P21579 | low | 2K4A | A | B | Transient | Transient | |
| P61328_P21579 | low | 2K4A | A | B | Transient | Transient | |
| P61020_O75116 | low | 2V55 | A | B | Transient | Transient | |
| P61587_P22612 | low | 2V55 | C | D | Transient | Transient | |
| P61587_P31749 | low | 2V55 | C | D | Transient | Transient | |
| P61587_P31751 | low | 2V55 | C | D | Transient | Transient | |
| Q04759_P61587 | low | 2V55 | C | D | Transient | Transient | |
| P47736_O14807 | low | 3BRW | B | D | Transient | Transient | |
| P47736_O14966 | low | 3BRW | B | D | Transient | Transient | |
| P47736_O95755 | low | 3BRW | B | D | Transient | Transient | |
| P47736_P0C0E4 | low | 3BRW | B | D | Transient | Transient | |
| P59190_P47736 | low | 3BRW | B | D | Transient | Transient | |
| P61018_P47736 | low | 3BRW | B | D | Transient | Transient | |
| P61587_P47736 | low | 3BRW | B | D | Transient | Transient | |
| P62491_P47736 | low | 3BRW | B | D | Transient | Transient | |
| P60709_P13796 | low | 3BYH | A | B | Transient | Transient | |
| P62736_P13796 | low | 3BYH | A | B | Transient | Transient | |
| P63261_P13796 | low | 3BYH | A | B | Transient | Transient | |
| P63267_P13796 | low | 3BYH | A | B | Transient | Transient | |
| P68032_P13796 | low | 3BYH | A | B | Transient | Transient | |
| P68133_P13796 | low | 3BYH | A | B | Transient | Transient | |
| P22891_O75635 | low | 3F1S | A | B | Transient | Transient | |
| P22891_O75830 | low | 3F1S | A | B | Transient | Transient | |
| P22891_P01009 | low | 3F1S | A | B | Transient | Transient | |
| P29508_P22891 | low | 3F1S | A | B | Transient | Transient | |
| P29622_P22891 | low | 3F1S | A | B | Transient | Transient | |
| P35237_P22891 | low | 3F1S | A | B | Transient | Transient | |
| P36955_P22891 | low | 3F1S | A | B | Transient | Transient | |
| P48594_P22891 | low | 3F1S | A | B | Transient | Transient | |
| P50454_P22891 | low | 3F1S | A | B | Transient | Transient | |
| P16144_A1L4K1 | low | 3F7Q | A | B | Transient | Transient | |
| P16144_O15197 | low | 3F7Q | A | B | Transient | Transient | |
| P16144_O60469 | low | 3F7Q | A | B | Transient | Transient | |
| P16144_P10586 | low | 3F7Q | A | B | Transient | Transient | |
| P16144_P13591 | low | 3F7Q | A | B | Transient | Transient | |
| P42701_P16144 | low | 3F7Q | A | B | Transient | Transient | |
| P54762_P16144 | low | 3F7Q | A | B | Transient | Transient | |
| Q00872_P16144 | low | 3F7Q | A | B | Transient | Transient | |
| P30281_P22674 | low | 3G33 | B | D | Obligatory | Transient | |
| P02675_O15123 | low | 3H32 | B | C | Obligatory | Transient | |
| P02675_O43827 | low | 3H32 | B | C | Obligatory | Transient | |
| P02675_O95841 | low | 3H32 | B | C | Obligatory | Transient | |
| P22105_P02675 | low | 3H32 | B | C | Transient | Transient | |
| P24821_P02675 | low | 3H32 | B | C | Transient | Transient | |
| P55083_P02675 | low | 3H32 | B | C | Transient | Transient | |
| P01579_A6NHT5 | verylow | 1FG9 | A | B | Transient | Obligatory | |
| P01579_O14627 | verylow | 1FG9 | A | B | Transient | Obligatory | |
| P01579_O43364 | verylow | 1FG9 | A | B | Transient | Obligatory | |
| P09016_P01579 | verylow | 1FG9 | A | B | Transient | Obligatory | |
| P0C7M4_P01579 | verylow | 1FG9 | A | B | Transient | Obligatory | |
| P31249_P01579 | verylow | 1FG9 | A | B | Transient | Obligatory | |
| P31273_P01579 | verylow | 1FG9 | A | B | Transient | Obligatory | |
| P31275_P01579 | verylow | 1FG9 | A | B | Transient | Obligatory | |
| O95972_O00622 | verylow | 1KLA | A | B | Transient | Obligatory | |
| P09529_O00622 | verylow | 1KLD | A | B | Transient | Obligatory | |
| O60499_O43680 | verylow | 1LR1 | A | B | Transient | Transient | |
| O60784_O43680 | verylow | 1LR1 | A | B | Obligatory | Transient | |
| P27487_P13798 | verylow | 1PFQ | A | B | Obligatory | Transient | |
| P27487_P22760 | verylow | 2BGN | A | B | Obligatory | Transient | |
| P02790_A6NL71 | verylow | 2F91 | A | B | Obligatory | Transient | |
| P08311_O60381 | verylow | 2F91 | A | B | Obligatory | Transient | |
| P10144_O60381 | verylow | 2F91 | A | B | Obligatory | Transient | |
| P14210_P12755 | verylow | 2F91 | A | B | Obligatory | Transient | |
| P26927_P02458 | verylow | 2F91 | A | B | Obligatory | Transient | |
| P35030_P14780 | verylow | 2F91 | A | B | Obligatory | Transient | |
| P40313_P14780 | verylow | 2F91 | A | B | Obligatory | Transient | |
| P30043_P28845 | verylow | 2IRW | A | B | Obligatory | Obligatory | |
| P29034_A2RU54 | verylow | 2JTT | A | B | Transient | Obligatory | |
| P33763_A2RU54 | verylow | 2JTT | A | B | Transient | Obligatory | |
| P33764_A2RU54 | verylow | 2JTT | A | B | Transient | Obligatory | |
| P33764_P09629 | verylow | 2JTT | A | B | Transient | Obligatory | |
| P33764_P17509 | verylow | 2JTT | A | B | Transient | Obligatory | |
| P33764_P31267 | verylow | 2JTT | A | B | Transient | Obligatory | |
| P33764_P31268 | verylow | 2JTT | A | B | Transient | Obligatory | |
| P29377_P23297 | verylow | 2KBM | A | B | Transient | Obligatory | |
| P32927_P21709 | verylow | 2RBL | A | B | Transient | Obligatory | |
| P42768_A4D1E9 | verylow | 1CEE | A | B | Obligatory | Transient | |
| P01579_O60479 | verylow | 1FG9 | A | B | Transient | Obligatory | |
| P39905_P01215 | verylow | 1HCN | A | B | Obligatory | Obligatory | |
| P36969_P32119 | verylow | 1N8J | G | H | Obligatory | Obligatory | |
| P11766_O95825 | verylow | 1U3T | A | B | Obligatory | Transient | |
| O95968_O43439 | verylow | 1UTR | A | B | Transient | Obligatory | |
| O95968_O75081 | verylow | 1UTR | A | B | Obligatory | Obligatory | |
| P05114_O43312 | verylow | 2D1L | A | B | Obligatory | Obligatory | |
| P10644_O15347 | verylow | 2EZW | A | B | Obligatory | Transient | |
| P10644_P0C6E5 | verylow | 2EZW | A | B | Transient | Transient | |
| P08311_O95503 | verylow | 2F91 | A | B | Transient | Transient | |
| P10144_O95503 | verylow | 2F91 | A | B | Transient | Transient | |
| P26927_P02751 | verylow | 2F91 | A | B | Transient | Transient | |
| P45973_A6NL71 | verylow | 2F91 | A | B | Transient | Transient | |
| P45973_P03952 | verylow | 2F91 | A | B | Transient | Transient | |
| P45973_P15157 | verylow | 2F91 | A | B | Transient | Transient | |
| P45973_P20231 | verylow | 2F91 | A | B | Transient | Transient | |
| O75604_B2RTY4 | verylow | 2HD5 | A | B | Transient | Obligatory | |
| O75604_O15211 | verylow | 2HD5 | A | B | Obligatory | Obligatory | |
| P27352_O75604 | verylow | 2HD5 | A | B | Obligatory | Obligatory | |
| P46108_O94953 | verylow | 2HKN | A | B | Obligatory | Transient | |
| P00519_O94953 | verylow | 2HL3 | A | B | Transient | Transient | |
| P42684_A8MW92 | verylow | 2HL3 | A | B | Transient | Transient | |
| P40818_O43566 | verylow | 2IBI | A | B | Transient | Obligatory | |
| O00626_A8MW92 | verylow | 2JP1 | A | B | Obligatory | Transient | |
| O00626_O00213 | verylow | 2JP1 | A | B | Transient | Transient | |
| P08118_P02776 | verylow | 2JP1 | A | B | Transient | Transient | |
| P10720_P08118 | verylow | 2JP1 | A | B | Transient | Transient | |
| P46934_O00626 | verylow | 2JP1 | A | B | Transient | Transient | |
| P46934_P13500 | verylow | 2JP1 | A | B | Transient | Transient | |
| P31260_P29034 | verylow | 2JTT | A | B | Transient | Obligatory | |
| O00213_A6NI72 | verylow | 2NNT | B | C | Obligatory | Transient | |
| O00213_A8MVU1 | verylow | 2NNT | B | C | Transient | Transient | |
| O14776_A6NKC9 | verylow | 2NNT | A | B | Transient | Transient | |
| O14776_O00264 | verylow | 2NNT | A | B | Transient | Transient | |
| O14776_O00634 | verylow | 2NNT | A | B | Transient | Transient | |
| O43918_O14776 | verylow | 2NNT | A | B | Transient | Transient | |
| O60687_O00213 | verylow | 2NNT | B | C | Transient | Transient | |
| O60687_O14776 | verylow | 2NNT | A | B | Transient | Transient | |
| O75400_A6NI72 | verylow | 2NNT | B | C | Transient | Transient | |
| O75400_A8MVU1 | verylow | 2NNT | B | C | Transient | Transient | |
| O75400_O60687 | verylow | 2NNT | B | C | Transient | Transient | |
| O75400_O60880 | verylow | 2NNT | C | D | Transient | Transient | |
| O75534_O14776 | verylow | 2NNT | A | B | Transient | Transient | |
| O94776_O14776 | verylow | 2NNT | A | B | Transient | Transient | |
| O94813_O14776 | verylow | 2NNT | A | B | Transient | Transient | |
| O94813_O75400 | verylow | 2NNT | B | C | Transient | Transient | |
| O94833_O00213 | verylow | 2NNT | A | B | Transient | Transient | |
| O94833_O75400 | verylow | 2NNT | A | B | Transient | Transient | |
| O95243_O00213 | verylow | 2NNT | A | B | Transient | Transient | |
| O95243_O14776 | verylow | 2NNT | A | B | Transient | Transient | |
| O95243_O75400 | verylow | 2NNT | A | B | Transient | Transient | |
| O95793_O75400 | verylow | 2NNT | A | B | Transient | Transient | |
| P08567_O75400 | verylow | 2NNT | A | B | Transient | Transient | |
| P12757_O14776 | verylow | 2NNT | C | D | Transient | Transient | |
| P14316_O14776 | verylow | 2NNT | B | C | Transient | Transient | |
| P14598_O00213 | verylow | 2NNT | B | C | Transient | Transient | |
| P14598_O75400 | verylow | 2NNT | B | C | Transient | Transient | |
| P35555_O14776 | verylow | 2NNT | A | B | Transient | Transient | |
| P42677_O14776 | verylow | 2NNT | A | B | Transient | Transient | |
| P46937_O94833 | verylow | 2NNT | B | C | Transient | Transient | |
| P46937_O95704 | verylow | 2NNT | A | B | Transient | Transient | |
| P46937_O95931 | verylow | 2NNT | B | C | Transient | Transient | |
| O94810_O00628 | verylow | 2PBI | C | D | Transient | Obligatory | |
| O94810_O43818 | verylow | 2PBI | C | D | Obligatory | Obligatory | |
| P25815_O75335 | verylow | 2PRU | A | B | Obligatory | Obligatory | |
| P26447_O75335 | verylow | 2PRU | A | B | Obligatory | Obligatory | |
| O94953_A8MW92 | verylow | 3FJ5 | A | B | Obligatory | Transient | |
| P40818_P10398 | verylow | 3I3T | A | B | Transient | Obligatory | |

“Model ID” – the PrePPI index number of the predicted dimer model. “Probability”- the PrePPI prediction likelihood: hight, low, very low. “Template ID” – the PDB accession number of the template PrePPI used to model the dimer. “Template Chain IDs” – the chains PrePPI used to model the structure. “Model” – DynaFace prediction of the interface type of the model structure: obligatory vs. non-obligatory. “Template” – DynaFace prediction of the interface of the template.
