# Supplementary material for: DynaFace: Discrimination between Obligatory and Non-obligatory Protein-Protein Interactions Based on the Complex’s Dynamics
Source: PLoS Comput Biol. 2015 Oct 27;11(10):e1004461. doi: 10.1371/journal.pcbi.1004461 (PMC4623975; doi:10.1371/journal.pcbi.1004461)
Supplement: S3 Table — (DOCX) [file pcbi.1004461.s007.docx]

**S3 Table.** **Individual performance of each attribute for obligatory and non-obligatory complex structures in the dataset.**

|  | AR | A_a | P_a | N_a | A_s | P_s | N_s |
| --- | --- | --- | --- | --- | --- | --- | --- |
| Oblig Success | 37.68% | 86.96% | 46.38% | 68.12% | 33.33% | 97.10% | 99.28% |
| Nonoblig Success | 62.64% | 64.53% | 79.25% | 68.30% | 77.74% | 11.32% | 4.15% |
| Overall Success | 54.09% | 72.21% | 67.99% | 68.24% | 62.53% | 40.69% | 36.72% |
