# Supplementary material for: DynaFace: Discrimination between Obligatory and Non-obligatory Protein-Protein Interactions Based on the Complex’s Dynamics
Source: PLoS Comput Biol. 2015 Oct 27;11(10):e1004461. doi: 10.1371/journal.pcbi.1004461 (PMC4623975; doi:10.1371/journal.pcbi.1004461)
Supplement: S4 Table — The values of the attributes for two examples are presented, where attributes that exceed the threshold are highlighted in bold. PDB IDs 1QU7 and 2SIC are predicted to be obligatory and non-obligatory, respectively, as they should. (DOCX) [file pcbi.1004461.s008.docx]

**S4 Table.** **The threshold value for the seven dynamic attributes used in a DynaFace calculations.**

|  | **AR** | **A_a** | **P_a** | **N_a** | **A_s** | **P_s** | **N_s** | **D** |
| --- | --- | --- | --- | --- | --- | --- | --- | --- |
| Threshold value for monomers with large (>65 residues) subunits | 0.0500 | -0.1230 | 0.1400 | -0.1200 | -0.1900 | 0.5900 | -0.6000 |  |
| Value for 1QU7 | 0.0045 | **-0.0086** | 0.2587 | -0.2170 | **0.0150** | **0.5402** | **-0.4121** | **4** |
| Value for 2SIC | 0.0131 | -0.1421 | **0.1254** | -0.1200 | -0.3895 | **0.4257** | **-0.3355** | **3** |

The values of the attributes for two examples are presented, where attributes that exceed the threshold are highlighted in bold. PDB IDs 1QU7 and 2SIC are predicted to be obligatory and non-obligatory, respectively, as they should.
