# Supplementary material for: DynaFace: Discrimination between Obligatory and Non-obligatory Protein-Protein Interactions Based on the Complex’s Dynamics
Source: PLoS Comput Biol. 2015 Oct 27;11(10):e1004461. doi: 10.1371/journal.pcbi.1004461 (PMC4623975; doi:10.1371/journal.pcbi.1004461)
Supplement: S5 Table — (DOCX) [file pcbi.1004461.s009.docx]

**S5 Table. The dynamic building units, structural units and hinge residues of an example obligomer: The homodimeric cytoplasmic domain of the serine chemotaxis receptor (1QU7 [**[**49**](#_ENREF_49)**]).**

| **Slowest mode** | ***Hinge residues*** | Chain A: HIS328/LEU329, THR450/ARG451 |
| --- | --- | --- |
|  |  | Chain B: HIS328/LEU329, ARG451/VAL452 |
|  | ***Dynamic structural domains*** | A:294-328, A:451-520/B:300-328, B:452-520 |
|  |  | A:329-450/B:329-451 |
| **Second Slowest mode** | ***Hinge residues*** | Chain A: ASP353/ILE354, GLY426/LYS427, VAL483/THR484 |
|  |  | Chain B: ASP353/ILE354, GLY426/LYS427 |
|  | ***Dynamic structural domains*** | A:294-353, A:427-483, B:300-353, B:427-520 |
|  |  | A:354-426/B:354-426, A:484-520 |
