# Supplementary material for: DynaFace: Discrimination between Obligatory and Non-obligatory Protein-Protein Interactions Based on the Complex’s Dynamics
Source: PLoS Comput Biol. 2015 Oct 27;11(10):e1004461. doi: 10.1371/journal.pcbi.1004461 (PMC4623975; doi:10.1371/journal.pcbi.1004461)
Supplement: S6 Table — (DOCX) [file pcbi.1004461.s010.docx]

**S6 Table.** **The dynamic building units, structural units and hinge residues of an example non-obligatory dimer: Subtilisin BPN' in complex with its streptomyces subtilisin inhibitor (2SIC [**[**52**](#_ENREF_52)**]).**

| **Slowest mode** | ***Hinge residues*** | Chain E: GLN275 |
| --- | --- | --- |
|  | ***Dynamic structural domains*** | E:1-275 |
|  |  | I:7-113 |
| **Second Slowest mode** | ***Hinge residues*** | Chain E: SER24/ASN25, VAL150/ALA151 |
|  |  | Chain I: HIS43/PRO44, PRO72/MET73, ASN99/GLU100 |
|  | ***Dynamic structural domains*** | E:1-24, E:151-275/I:7-43, I:73-99 |
|  |  | E:25-150, I:44-72, I:100-113 |
