# Supplementary material for: DynaFace: Discrimination between Obligatory and Non-obligatory Protein-Protein Interactions Based on the Complex’s Dynamics
Source: PLoS Comput Biol. 2015 Oct 27;11(10):e1004461. doi: 10.1371/journal.pcbi.1004461 (PMC4623975; doi:10.1371/journal.pcbi.1004461)
Supplement: S7 Table — (DOCX) [file pcbi.1004461.s011.docx]

**S7 Table.** **The comparison of the predictions of the servers; PQS, PINS, NOXclass, PISA and DynaFace on the crystal dataset by [**[**53**](#_ENREF_53)**].**

| ***PDB*** | ***PQS*** | ***PINS*** | ***NOXCLASS*** | ***PISA*** | ***PITPRED*** |
| --- | --- | --- | --- | --- | --- |
| 1a3y | Crystal | Crystal | Crystal | Monomeric | Non-obligatory |
| 1a7v | Crystal | Crystal | Crystal | Monomeric | Non-obligatory |
| 1ae9 | Hexamer | Hexohexamer | Crystal | Monomeric | Non-obligatory |
| 1afk | Crystal | Crystal | Crystal | No result | Non-obligatory |
| 1ag9 | Crystal | Homodimer | Crystal | Dimer | Non-obligatory |
| 1ajk | Crystal | Crystal | Crystal | Monomeric | Non-obligatory |
| 1aq0 | Crystal | Homodimer | Crystal | Monomeric | Non-obligatory |
| 1aqz | Crystal | Crystal | Crystal | Monomeric | Non-obligatory |
| 1b80 | Tetrameric | Crystal | Crystal | Monomeric | Non-obligatory |
| 1bc2 | Crystal | Homodimer | Biological | Monomeric | Non-obligatory |
| 1bin | Crystal | Crystal | Crystal | No result | Non-obligatory |
| 1bkz | Crystal | Homodimer | Crystal | Monomeric | Non-obligatory |
| 1byo | Homotetramer | Crystal | Crystal | Monomeric | Non-obligatory |
| 1c02 | Dimeric | Homohexamer | Biological | Dimer | Non-obligatory |
| 1cfy | Crystal | Crystal | Crystal | Monomeric | Non-obligatory |
| 1cqx | Homodimer | Homodimer | Crystal | Dimer | Non-obligatory |
| 1dsu | Crystal | Homodimer | Crystal | Monomeric | Non-obligatory |
| 1dxm | Dimeric | Homodimer | Crystal | Monomeric | Non-obligatory |
| 1dys | Dimeric | No result | Crystal | Monomeric | Non-obligatory |
| 1dz4 | Crystal | Crystal | Crystal | Monomeric | Non-obligatory |
| 1elp | Crystal | Crystal | Crystal | Monomeric | Non-obligatory |
| 1epa | Crystal | Homodimer | Crystal | Dimer | Non-obligatory |
| 1fgk | Crystal | Homodimer | Crystal | Monomeric | Non-obligatory |
| 1fvk | Crystal | Homodimer | Crystal | Monomeric | Non-obligatory |
| 1gar | Dimeric | Crystal | Crystal | Dimer | Non-obligatory |
| 1hrn | Hexameric | No result | Crystal | Hexamer | Non-obligatory |
| 1i4g | Crystal | Crystal | Biological | Monomeric | Non-obligatory |
| 1ihb | Crystal | Crystal | Crystal | Monomeric | Non-obligatory |
| 1ilr | Crystal | Homodimer | Biological | Monomeric | Non-obligatory |
| 1jfr | Crystal | Crystal | Crystal | Monomeric | Non-obligatory |
| 1kpt | Crystal | Homodimer | Crystal | Monomeric | Non-obligatory |
| 1kwa | Crystal | Crystal | Crystal | Tetramer | Non-obligatory |
| 1mpg | Crystal | Crystal | Crystal | Dimer | Non-obligatory |
| 1mss | Dimeric | Homodimer | Biological | Dimer | Obligatory |
| 1mwc | Crystal | No result | Crystal | Monomeric | Non-obligatory |
| 1naw | Tetrameric | Homotetramer | Crystal | Tetramer | - |
| 1ome | Crystal | Homododecamer | Biological | No result | Non-obligatory |
| 1pbg | Crystal | Crystal | Crystal | No result | - |
| 1qci | Dimeric | Crystal | Crystal | Monomeric | Non-obligatory |
| 1qha | Crystal | Homodimer | Biological | Monomeric | Obligatory |
| 1rb3 | Dimeric | Crystal | Crystal | Monomeric | Non-obligatory |
| 1rge | Crystal | Crystal | Crystal | No result | Non-obligatory |
| 1shk | Tetrameric | Homodimer | Crystal | Dimer | Non-obligatory |
| 1sw6 | Crystal | Homodimer | Biological | Dimer | Non-obligatory |
| 1tht | Crystal | Homodimer | Crystal | Dimer | Non-obligatory |
| 1toa | Dimeric | Crystal | Crystal | Dimer | Non-obligatory |
| 1vlz | Dimeric | Crystal | Crystal | Monomeric | Non-obligatory |
| 1xca | Dimeric | Crystal | Biological | Dimer | Non-obligatory |
| 1xgs | Dimeric | Homodimer | Biological | Monomeric | Non-obligatory |
| 1ygh | Crystal | Homopentamer | Crystal | Monomeric | - |
| 256b | Crystal | Crystal | Crystal | Monomeric | Non-obligatory |
| 2atj | Crystal | Crystal | Crystal | Monomeric | Non-obligatory |
| 2bc2 | Crystal | Crystal | Crystal | No result | Non-obligatory |
| 2bls | Crystal | Crystal | Crystal | No result | Non-obligatory |
| 2erc | Hexameric | Crystal | Crystal | Dimer | - |
| 2g3p | Dimeric | Homodimer | Biological | Dimer | Obligatory |
| 2scp | Dimeric | Homodimer | Biological | No result | Non-obligatory |
| 2tps | Tetrameric | Crystal | Biological | Monomeric | Non-obligatory |
| 2ugi | Dimeric | No result | Biological | Dimer | Non-obligatory |
| 3ng1 | Crystal | Crystal | Crystal | Monomeric | Non-obligatory |
| 3pmg | Crystal | Crystal | Crystal | Monomeric | Non-obligatory |
| 5tss | Crystal | Crystal | Crystal | No result | Non-obligatory |
| 830c | Tetrameric | Crystal | Biological | Tetramer | Non-obligatory |
