# Supplementary material for: DynaFace: Discrimination between Obligatory and Non-obligatory Protein-Protein Interactions Based on the Complex’s Dynamics
Source: PLoS Comput Biol. 2015 Oct 27;11(10):e1004461. doi: 10.1371/journal.pcbi.1004461 (PMC4623975; doi:10.1371/journal.pcbi.1004461)
Supplement: S8 Table — (DOCX) [file pcbi.1004461.s012.docx]

**S8 Table. The dynamic building units, structural units and hinge residues of the structural model P09529_P01137 based on the template NMR structure TGF-B1 (1KLD[**[**59**](#_ENREF_59)**]).**

| **Slowest mode** | ***Hinge residues*** | Chain A: GLU333/GLY334, CYS372/ILE373, CYS404/GLY405 |
| --- | --- | --- |
|  |  | Chain B: GLY46/PRO47, CYS77/CYS78 |
|  | ***Dynamic structural domains*** | A:301-333, A:373-404, B:47-77 |
|  |  | A:334-372, A:405-406, B:1-46, B:78-112 |
| **Second Slowest mode** | ***Hinge residues*** | Chain A: PHE309/ILE310, TYR327/TYR328, THR379/MET380, ASP395/VAL396 |
|  |  | Chain B: LEU20/TYR21, ALA41/ASN42, GLU84/PRO85, MET104/ILE105 |
|  | ***Dynamic structural domains*** | A:301-309, A:328-379, A:380-395, B:21-41, B:85-104 |
|  |  | A:310-327, A:396-406, B:1-20, B:42-84, B:105-112 |
