# Supplementary material for: DynaFace: Discrimination between Obligatory and Non-obligatory Protein-Protein Interactions Based on the Complex’s Dynamics
Source: PLoS Comput Biol. 2015 Oct 27;11(10):e1004461. doi: 10.1371/journal.pcbi.1004461 (PMC4623975; doi:10.1371/journal.pcbi.1004461)
Supplement: S1 Method — (DOCX) [file pcbi.1004461.s013.docx]

## METHOD: Server

**Dynamic scoring.** Formally, the variables A, P and N can be expressed as follows:

$$A=\frac{\sum_{i\in S_{1}} \sum_{j\in S_{2}} C_{\mathrm{ij}}}{\left| S_{1} \right|\left| S_{2} \right|}$$

$$P=\frac{\sum_{i\in S_{1}} \sum_{j\in S_{2}} \left\{ \begin{aligned} C_{\mathrm{ij}}, C_{\mathrm{ij}}>0 \\ 0, else \end{aligned} \right.}{\left| \left\{ i\in S_{1}, j\in S_{2}|C_{\mathrm{ij}}>0 \right\} \right|}$$

$$N=\frac{\sum_{i\in S_{1}} \sum_{j\in S_{2}} \left\{ \begin{aligned} C_{\mathrm{ij}}, C_{\mathrm{ij}}<0 \\ 0, else \end{aligned} \right.}{\left| \left\{ i\in S_{1}, j\in S_{2}|C_{\mathrm{ij}}<0 \right\} \right|}$$

where S_1_ and S_2_ are subunits 1 and 2, respectively. A subunit may refer to a single chain or more than one chain in a given complex structure. $C_{\mathrm{ij}}$ refers to the correlation value between residues i and j in the given mode.
